# Genome-wide identification and dynamic transcriptome profiling of the DYW-type PPR family across greening of chlorotic leaves in pear (*Pyrus pyrifolia*)

**DOI:** 10.3389/fpls.2026.1767760

**Published:** 2026-01-29

**Authors:** Liqing Lu, Haiqi Zhang, Zixian Zha, Xueqian Wang, Na Ma, Chunyan Liu, Yiliu Xu, Zhenghui Gao, Yongjie Qi

**Affiliations:** Key Laboratory of Horticultural Crop Germplasm Innovation and Utilization (Co-Construction by Ministry and Province), Institute of Horticulture Anhui Academy of Agricultural Sciences, Hefei, China

**Keywords:** pear, DYW-type PPR, chloroplast development, transcriptome, *PpPPR115*

## Abstract

**Introduction:**

Pear (*Pyrus pyrifolia*) chlorotic leaves severely impair photosynthesis and the accumulation of photosynthetic products, primarily due to abnormal chloroplast development. DYW-type PPR proteins play a crucial role in regulating chloroplast development and maintaining structural integrity.

**Methods:**

To comprehensively characterize the involvement of DYW-type PPR proteins in pear leaf and chloroplast development, we performed a genome-wide identification of 129 DYW-type PPR proteins in pears and systematically analyzed their sequence diversity, protein domain architecture, and evolutionary relationships.

**Results:**

Compared with the wild-type ‘Chuxialv’, the ‘Chuxialv’ bud mutant exhibits reduced chlorophyll and ferrous ion content, along with disrupted chloroplast ultrastructure in leaves. Using this paired material, we conducted high-depth whole-genome resequencing to identify structural variations within the DYW-type PPR gene family. Furthermore, RNA-seq was performed on leaf samples from yellow to green, spanning five distinct developmental stages to construct a temporal expression profile of DYW-type PPR genes. Six DYW-type PPR genes exhibiting differential expression were identified, and protein-protein interaction network analysis of them, coupled with functional enrichment analysis, provided the underlying regulatory mechanism in chloroplast development and photosynthesis. Coexpression and functional regulatory networks of DYW-type PPR genes were constructed by integrating weighted gene co-expression network analysis with gene ontology enrichment analysis. Notably, only the coexpression module centered on *PpPPR115* was enriched in photosynthesis-related biological processes. Furthermore, the MYB transcription factor binding motif was identified in the promoter region of *PpPPR115*. Six MYB transcription factors down-regulated in the CM1 compared with CL1 were excavated. Dual-luciferase reporter assays and yeast one-hybrid assays confirmed that *PpMYB102* may act as a key upstream regulator of *PpPPR115*.

**Discussion:**

In conclusion, this study elucidated the structural variations and dynamic expression patterns of the DYW-type PPR gene associated with chlorotic leaves in pears, offering novel insights and potential regulatory pathways relevant to chloroplast development and the transformation of chlorotic leaves to green in pears.

## 1 Introduction

The pentatricopeptide repeat (PPR) protein family is one of the largest protein families in plants, and its members play fundamental roles in the post-transcriptional regulation of mitochondria and chloroplast genes (Lurin et al., 2004), influencing photosynthesis, respiration, and response processes to environmental stress. PPR proteins typically contain 2–30 tandem repetitive domains, each composed of 31–36 amino acid residues. Based on the composition of these residues, the domains are classified into P, L, and S models. PPR proteins composed entirely of P models are categorized as P-type PPR proteins. In contrast, those with alternating P, L, and S models are categorized as typical PLS-type PPR proteins. Additionally, the C-terminal region of the PLS subfamily members includes E, E+, and DYW domains. Among them, proteins with the DYW domain are further subdivided into DYW-type PPR proteins (Cheng et al., 2016).

Owing to the unique catalytic activity of the DYW domain, DYW-type PPR family genes tend to exhibit more diverse and specialized biological functions, particularly in C-to-U editing. C-to-U editing is an error correction approach which critical for the normal translation of organelle proteins and plant growth. Typically, the C-to-U editing process involves collaborative efforts of multiple protein editing factors. The PPR proteins bind to RNA with high specificity, while the DYW domain in these proteins exerts deaminase activity to mediate C-to-U editing (Zhang et al., 2023). Required for accD RNA editing 1 (RARE), a DYW-type PPR protein, stabilizes plastid membrane development under heat stress by participating in C-to-U editing at the C749 site of chloroplast accD transcripts in Arabidopsis (Huang et al., 2023). Previously, based on big data analysis of 2.25 million PPR motifs, a DYW domain model for catalytic editing of C-to-U was constructed (Gutmann et al., 2020). This model provided valuable insights into the relationship between various DYW-type PPR domains and the C-to-U editing efficiency. Notably, truncation or deletion of the DYW domain has been shown not to affect the editing efficiency of PPR proteins in some species. For example, cabbage *yellow-green leaf 2* (*BoYgl-2*), which encodes P-type PPR proteins without the DYW domain, is involved in the C-to-U editing events in chloroplasts. Mutants of this gene exhibited chlorotic leaves and reduced chlorophyll content (Zhang et al., 2024). However, the DYW domains of *quintuple editing factor 1* (*QED1*) and *RARE1* have been proven essential for their C-to-U editing abilities (Liu et al., 2023; Wagoner et al., 2015). These findings indicated that the functional conservation of the DYW domain across different species warrants further investigation. Furthermore, the editing of other sites is also within the scope of DYW-type PPR proteins. For instance, *OsPGL3A*, which contains typical DYW domains, is located in chloroplasts and participates in the editing of rps8–182 and rpoC2–4106 loci as well as the splicing of *ycf3–1* in rice (Xu et al., 2024). *AtECB2*, belonging to the DYW-type PPR family, is responsible for editing plastid *accD* and *ndhF* transcripts and collaborates with the hydroxymethylbilane synthase (HEMC) and multiple organelle RNA editing factor (MORF) family genes for plastid RNA editing. Mutations in this gene lead to delayed greening of *Arabidopsis thaliana* leaves (Cao et al., 2011; Huang et al., 2017), revealing the close association of DYW-type PPR proteins with chloroplast development and chlorotic leaves.

In addition to the DYW-type PPR proteins, other subtypes of PPR proteins are also necessary for the growth and development of plants. Chloroplasts possess small, independent genomes, and the post-transcriptional RNA editing process is crucial for chloroplast development and the expression of their genes, which serve as important regulatory factors for chlorotic leaves. In rice, the PLS-type PPR protein OsASL, which contains 12 PPR repeat domains, participates in the intron splicing and RNA editing of plastid-encoded RNA polymerase (PEP)-coding genes, including *atpF*, *ndhA*, *rpl2*, *rps12*, and *ndhB* (Li et al., 2024). In plants containing the *ylws* mutation of P-type PPR proteins, chloroplasts show developmental defects attributed to abnormal intron cleavage of *atpF*, *ndhA*, *rpl2*, and *rps12* (Lan et al., 2023). The P-type PPR protein ECD2 is involved in the splicing of type II introns, and its down-regulation impacts ribosome accumulation in the chloroplast, resulting in cotyledon albinism (Wang et al., 2021). Similarly, *OsWAL3*, a gene encoding a PLS-type PPR protein, is also involved in type II intron splicing, and its deletion induces the albinism phenotype in rice leaves (Lv et al., 2022). *Regulation factor of chloroplast development 1* (*RFCD1*) knockout mutants are lethal, and the related interference lines exhibit downregulation of PEP-encoding genes, resulting in chlorotic leaves and abnormal chloroplast development (Tan et al., 2025). *GhCTF1*, which contains only two PPR motifs, also plays a pivotal role in chloroplast development and is involved in the intron splicing of *YCF2–3* and PEP subunit rpoC1 in cotton (Huo et al., 2024). The knockout of *GhCTEF2*, a PLS-type PPR protein in cotton, leads to macular formation in leaves and abnormal chloroplast structure, which correlates with the decreased transcriptional levels of PEP-dependent genes and light-harvesting complex (LHC) II-T complex protein (He et al., 2025). These studies highlighted the intimate relationship between PPR proteins and chlorotic leaves.

PPR proteins are also involved in RNA editing, processing and translation in mitochondria. In maize, PPR proteins DEK2 and DEK56 are responsible for transcript editing in mitochondria and mediate embryo and endosperm development (Qi et al., 2017; Zang et al., 2024). The small PPR protein SPR2 is essential for intron cleavage in maize mitochondria, and its mutation hinders embryo and endosperm development (Cao et al., 2022). This finding indicated that SPR2, a core subunit of the splicing complex, might exert its biological functions by interacting with the substrate recognized by the PPR protein. Some PPR proteins lacking typical DYW domains, such as the E+ type CWM1, can still perform RNA editing on mitochondria *nad5* transcripts by recruiting DYW domains to form editing complexes (Oldenkott et al., 2020). The functions of DYW-type PPR proteins across different species exhibit varying degrees of conservation, which is likely attributable to differences in structural features and the influence of natural selection.

Chlorotic leaf is a common and challenging problem in pear production, and the mechanisms underlying the regulation of pear chlorotic leaves and chloroplast development by DYW-type PPR proteins remain elusive. In this study, we identified 129 DYW-type PPR genes in pear and analyzed their evolutionary relationships and domain characteristics. The ‘Chuxialv’ pear and its bud mutation material were subjected to whole-genome resequencing and RNA-seq. Several DYW-type PPR structural variation sites related to chlorotic leaves were identified through whole-genome resequencing. The expression profiles and protein–protein interaction networks of the DYW-type PPR genes across five distinct developmental stages spanning the yellowing-greening transition were revealed via RNA-seq. Coexpression and functional enrichment networks of the DYW-type PPR genes were constructed through weighted gene co-expression network analysis (WGCNA). The strong correlation between *PpPPR115* expression and pear chlorotic leaves was further validated, and the upstream regulatory factor *PpMYB102* was shown to positively regulate the expression of *PpPPR115*. In summary, the DYW-type PPR protein family in pear was characterized, and the relationship between DYW-type PPR and pear chlorotic leaves was elucidated on the basis of both structural variations and expression changes. Notably, the *PpMYB102*-*PpPPR115* module might play an underlying regulatory role in the transformation of pear chlorotic leaves to green.

## 2 Materials and methods

### 2.1 Plant materials

**Figure 1:**
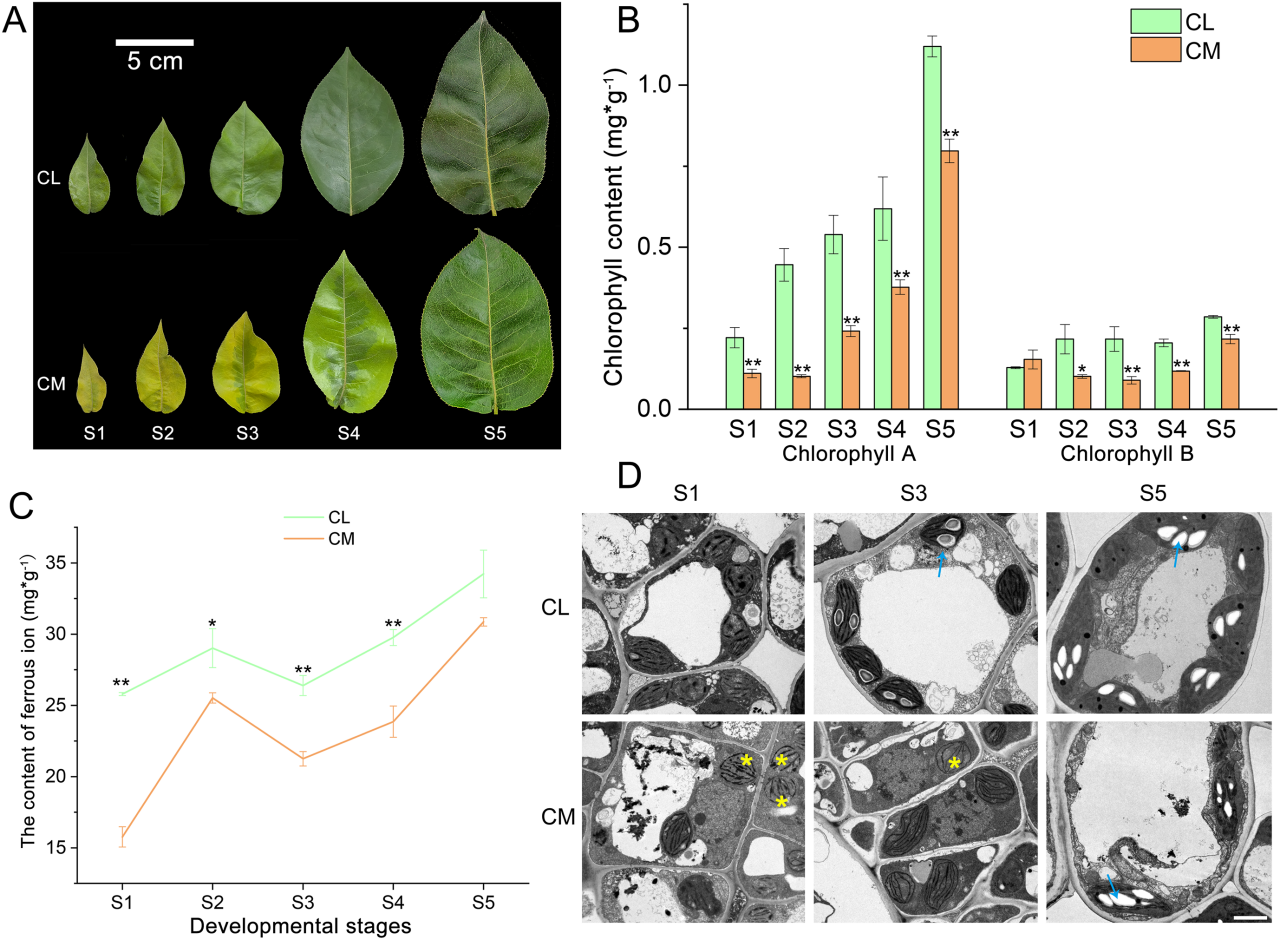
Leaf physiological characteristics of ‘Chuxialv’ pear (CL) and its bud mutation (CM). **(A)** Leaf morphology of CLs and CMs across five developmental stages. Bar: 5 cm. **(B)** Chlorophyll A and chlorophyll B contents of CLs and CMs across five developmental stages. Statistical significance was determined by an independent samples t-test (*P < 0.05; **P < 0.01). **(C)** Content of ferrous ion in leaves at different developmental stages. Statistical significance was determined by an independent samples t-test (*P < 0.05; **P < 0.01). **(D)** Chloroplast ultrastructure of CLs and CMs. The yellow asterisks indicate structurally abnormal chloroplasts; the blue arrows indicate starch grains. Bar: 2 μm.

The sample morphologies at different developmental stages are shown in **Figure 1A**. The leaves of ‘Chuxialv’ pear (*Pyrus pyrifolia*, CL, as a control) are green throughout the entire developmental stage. The leaves of its bud mutant (*P. pyrifolia*, CM) pear are yellow in the early development stage, but naturally turn green in the later stage. The leaf expansion stage was defined as 0 days, while the sampling times of CL1/CM1 (S1), CL2/CM2 (S2), CL3/CM3 (S3), CL4/CM4 (S4) and CL5/CM5 (S5) were 6, 12, 18, 24 and 30 days, respectively. All plant materials were collected from the experimental base located in Lu’an city, Anhui Province, China, and the composition of the cultivation soil and fertilizer was exactly the same. The pear cultivar ‘Chuxialv’ was developed by Prof. Zebin Shi from the Institute of Horticulture, Zhejiang Academy of Agricultural Sciences. The bud mutant of ‘Chuxialv’ pear was identified by Yongjie Qi from the Institute of Horticulture, Anhui Academy of Agricultural Sciences.

### 2.2 Identification of DYW-type PPR subfamily genes

The *P. pyrifolia* genome sequences were downloaded from https://download.cncb.ac.cn/gwh/Plants/Pyrus_pyrifolia_Pyrus_pyrifolia_cultivar_Cuiguan_GWHBAOS00000000. The hidden Markov model (HMM) file of the DYW-type PPR subfamily (PF14432) was downloaded from InterPro and scanned for the conserved domain of the DYW-type PPR family to obtain candidate family members. The integrity of the DYW-type PPR functional domains was determined using SMART (http://smart.embl-heidelberg.de/) (Letunic et al., 2020) and CDD (https://www.ncbi.nlm.nih.gov/cdd/) (Wang et al., 2023).

### 2.3 Phylogenetic tree construction, gene structure analysis, and biochemical characterization

A phylogenetic tree of the DYW-type PPR family members was constructed using the MEGA7 (v7.0.26) maximum likelihood method with 1000 bootstrap replicates. The “Motif Discovery module” on the MEME website was used for protein domain identification, with “Advanced options” as the default parameter (Bailey et al., 2015). The 2-kb sequence upstream of the 5’ UTR was selected as the promoter sequence, with the binding motifs predicted using the PlantCARE website and visualized using the “Gene Structure View” of the TBtools plugin (Chen et al., 2020; Lescot et al., 2002). Cell-PLoc 2.0 (http://www.csbio.sjtu.edu.cn/bioinf/Cell-PLoc-2/) was used to predict the subcellular localization states of the DYW-type PPR gene family members (Chou and Shen, 2008). ExPASy (http://web.expasy.org/compute_pi/) was used to analyze the isoelectric points and molecular weights of the proteins (Bjellqvist et al., 1993).

### 2.4 Intraspecific collinearity analysis

The intraspecific collinearity analysis of the DYW-type PPR family was performed using “MCScanX”, “Text Merge for MCScanX” and “Text Transformat for micro-synteny view” plugin of TBtools. The “advanced circos” module was applied for the visualization of collinearity events. The Ka/Ks values of collinear events were calculated using Tbtools (Chen et al., 2020).

### 2.5 Determination of chlorophyll content

The pear leaves (0.2 g) were chopped and soaked in 95% ethanol for 24 hours. Subsequently, the filtrate was centrifuged at low speed for 5 minutes to remove the sediment, and the absorbance at 663 nm and 645 nm was measured using a microplate reader (Tecan Infinte E Plex). The Arnon formula was used to calculate the content of chlorophyll a and chlorophyll b, respectively.

### 2.6 Transmission electron microscope

The pear leaves from three developmental stages (S1, S3, S5) were collected and fixed with glutaraldehyde fixative for 4 hours at 4 °C. Fixed for another 2 hours in 1% osmium acid-0.1M PBS buffer. The tissue was dehydrated in gradient ethanol for 15 minutes each. Then, embedding overnight and polymerizing at 60°C for 48 hours were carried out. The embedded tissues were subjected to ultrathin sections and then stained with uranium-lead double staining (2% uranium acetate saturated aqueous solution and lead citrate for 15 minutes each) and observed under a transmission electron microscope.

### 2.7 Whole-genome resequencing

The leaves of CL and CM were subjected to genomic DNA extraction with the CTAB method. Library construction and quantification were performed after fragmenting high-quality genomic DNA. The 50x high-depth sequencing was performed using Combinatorial Probe-Anchor Synthesis to obtain the raw data. Clean data was obtained after data filtering and aligned to the reference genome using bwa mem (v0.7.17) (Vasimuddin et al., 2019), followed by filtering out low-quality and duplicate reads. SNPs/InDels were detected using GATK, and ANNOVAR was used to annotate the obtained variant sites.

### 2.8 RNA-sequencing

The leaf expansion stage was defined as 0 days, while the sampling times of CL1/CM1, CL2/CM2, CL3/CM3, CL4/CM4 and CL5/CM5 were 6, 12, 18, 24 and 30 days, respectively. High-quality RNAs from CL and CM leaves across five developmental stages (from yellowing to greening) were extracted, fragmented, and reverse-transcribed into cDNA with adapters for library construction and sequencing. The whole-genome raw data of the corresponding samples were obtained. After filtering, the clean data were generated and mapped to the *P. pyrifolia* (‘Cuiguan’) genome. The expression levels were normalized as fragments per kilobase of exon model per million mapped fragments (FPKM) value. Finally, the DESeq2 package (Love et al., 2014) was used to identify differential gene expression.

### 2.9 Construction and visualization of the protein-protein interaction network

A PPI regulatory network of DYW-type PPR proteins was constructed using STRING (Szklarczyk et al., 2023), with a screening threshold for binding strength set as 0.9. Cytoscape (Shannon et al., 2003) was used to visualize the PPI regulatory network. The node size reflects the degree number, and the edge width reflects the combined score between two nodes.

### 2.10 Functional enrichment analysis

The GO enrichment analysis was performed using the default parameters of KOBAS 3.0 (http://bioinfo.org/kobas/) (Bu et al., 2021) to obtain the functional annotations of the target gene sets. The two columns of data, GO term and corresponding P value, were input into the REVIGO website (http://revigo.irb.hr/) (Supek et al., 2011). The redundant GO terms were eliminated according to the default parameters (0.7). Finally, the non-redundant GO terms in the biological process category were retained. Cytoscape was used to visualize the data, with the log_10_ (number of enriched genes/total genes) and -log_10_ P values reflected in the circle size and color depth, respectively. The R package “upsetR” was used to visualize the intersection of genes among different biological processes (Gehlenborg, Nils, 2015).

### 2.11 Weighted gene co-expression network analysis

A total of 31,237 genes with a maximum FPKM value of no less than 0.3 across the ten samples were used to construct the WGCNA network using the WGCNA package (Langfelder and Horvath, 2008). Since the power curve did not meet expectations, the power value was set to 16 according to the official recommendation and the network was constructed accordingly. The minModuleSize and mergeCutHeight were set to 30 and 0.25, respectively.

### 2.12 Yeast one-hybrid

The yeast one-hybrid system and vectors were derived from a previous study (Lu et al, 2023). *PpPPR115* promoter sequence was ligated to the pAbAi plasmid digested by *Hind* III and *Kpn* I through homologous recombination. After being digested by *Bbs* I, the recombination plasmid was transferred to the Y1HGold strain, and the self-activation concentration of aureobasidin A (AbA) was screened. Subsequently, the GADT7 plasmid containing the *PpMYB102* sequence and the empty GADT7 plasmid were respectively transferred to the Y1HGold strain as the experimental group and the control group. The strains of the experimental and control groups were added to Leu-defective plates containing the corresponding AbA concentrations for self-activation. The interaction relationship between *PpPPR115* and *PpMYB102* was determined based on the growth status of yeast.

### 2.13 Dual-luciferase assay

The *PpPPR115* promoter was ligated to the pGreenII-0800 vector, cleaved by *Hind* III and *BamH* I. The complete CDS of *PpMYB102* was ligated to the 62SK vector cleaved by *Hind* III and *Kpn* I. After verification by Sanger sequencing, these two vectors were transferred into *Agrobacterium tumefaciens* GV3101. Subsequently, it was mixed at a ratio of pGreenII 0800: pGreenII 62SK = 3: 1 and injected into tobacco leaves. After two days of incubation in the dark, the leaves were treated with the dual one step luciferase reporter gene assay kit (Yeasen, 11405ES60) followed by fluorescence reading using a microplate reader (Tecan Infinite E Plex) with default settings. The pGreenII 62SK empty vector was used as a negative control.

### 2.14 Quantitative RT-PCR

The pear leaves were frozen in liquid nitrogen immediately after collection and subjected to RNA extraction with an RNA extraction kit (Tiangen, DP452) as instructed. Power SYBR Green PCR Master Mix (Thermo Fisher) was used to construct the PCR mixture. Gene expression analysis was performed using a CFX Connect Real-Time System (BIO-RAD) with three replicates. The relative expression levels of the genes were calculated using the 2^-ΔΔ^CT formula.

### 2.15 Data processing

Statistical significance was determined by an independent samples t-test (*P < 0.05; **P < 0.01). Statistical significance of Letter Notation was determined by Fisher’s LSD test and Duncan test (*P < 0.05; **P < 0.01). Three replicates are applied to each sample.

## 3 Results

### 3.1 Profiling analyses of the physiological morphology and indicators of ‘Chuxialv’ pear and its bud mutation

To investigate the physiological characteristics associated with the yellowing-greening transition in CM leaves (**Figure 1A**), we performed chlorophyll quantification, determination of the ferrous ion (Fe²^+^) content, and ultrastructural observation of chloroplasts. The chlorophyll A levels in CM were significantly lower than those in CL across all five developmental stages (**Figure 1B**). However, a gradual increase in chlorophyll A was observed in CM during S4 and S5, indicating a greening trend. The chlorophyll B levels differed only slightly between CL and CM, suggesting a secondary role in leaf development and greening (**Figure 1B**). The content of Fe²^+^ in CM remained consistently lower than that in CL throughout the developmental stages, but also exhibited a progressive increase, implying a strong correlation between Fe²^+^ availability and chlorophyll accumulation (**Figure 1C**). Furthermore, ultrastructural analysis revealed that CL exhibited normal and well-organized chloroplasts at S1, S3, and S5, with visible starch granules indicative of active photosynthesis (**Figure 1D**). In contrast, CM leaves displayed underdeveloped chloroplasts at S1 and S3, with starch granules absent until S5. These structural observations suggest impaired photosynthetic capacity in CM during early development, which progressively recovers as the leaves mature (S5).

### 3.2 Identification of DYW-type PPR gene family in Pyrus pyrifolia

**Figure 2:**
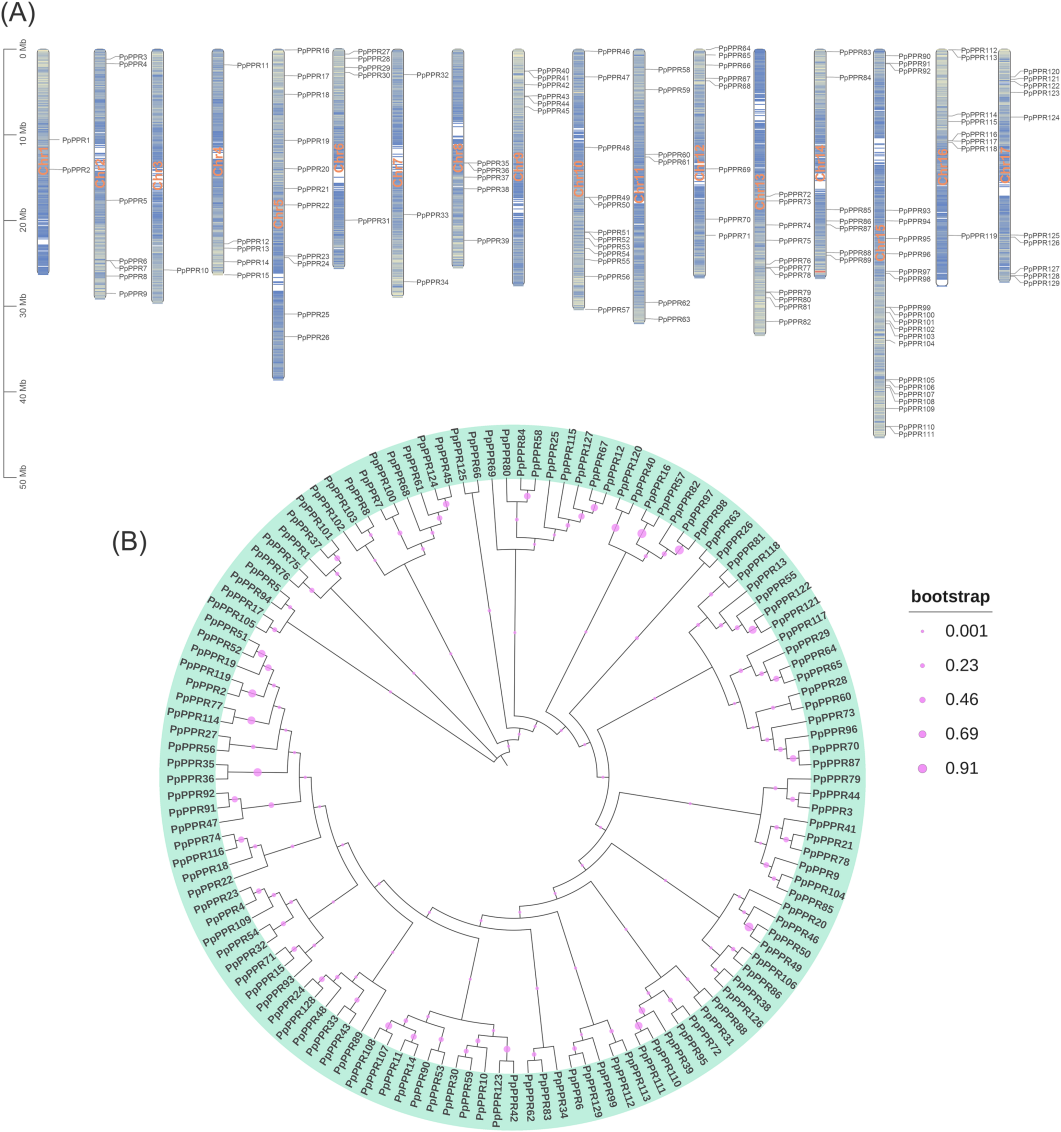
Chromosome distribution of DYW-type PPR genes and phylogenetic tree of corresponding proteins in pear. **(A)** Distribution of 129 DYW-type PPR genes on different chromosomes. The depth of blue represents the gene density on chromosomes. **(B)** Phylogenetic analysis of 129 DYW-type PPR proteins based on the maximum likelihood method (bootstrap = 1000). The bootstrap value of each sub-branch is reflected on the size of the pink dots.

An HMM search was employed with PF14432 to systematically identify DYW-type PPR family members in the pear genome. A total of 129 genes encoding proteins containing typical DYW-type PPR domains were identified (**Figure 2A**). These genes are distributed across 17 chromosomes in pear. Several family members, such as *PpPPR6*/*PpPPR7* and *PpPPR35*/*PpPPR36*, formed gene clusters in adjacent loci, which might be attributed to tandem gene replication events and similar functions. The DYW-type PPR genes also exhibited inconsistent distribution patterns across different chromosomes (**Supplementary Figure S1**). For instance, chr15, chr1 and chr3 harbor 22, two and one DYW-type PPR genes, respectively. A phylogenetic tree of the 129 DYW-type PPR genes was constructed using the maximum likelihood method to gain insights into the evolutionary landscape (**Figure 2B**). These genes were grouped into several subclusters based on their evolutionary distances. The bootstrap values ranged from 0.001 to 0.91, among which the bootstrap values of 19 branches were greater than 0.5, and 10 branches were greater than 0.7 (**Figure 2B**). The overall low bootstrap values indicate that the DYW-type gene family may have undergone rapid expansion and formed potential redundant genes. Gene pairs located adjacent to each other and clustered together on the chromosome, such as *PpPPR64*-*PpPPR65* and *PpPPR102*-*PpPPR103*, represent potential sites of gene replicate events. Some gene pairs, such as *PpPPR35*/*PpPPR36*, *PpPPR110*/*PpPPR111* and *PpPPR49*/*PpPPR50*, exhibit extremely high bootstrap values in the phylogenetic tree, suggesting that these gene pairs may have originated from a common gene evolution process and possess similar biological functions. In addition, the physicochemical properties and subcellular localization of these genes were characterized (**Supplementary Table S1**). The isoelectric point (pI) and molecular weight (MW) of the proteins encoded by these 129 genes ranged from 5.57 to 9.04 (average: 7.26) and 24987 to 174574 Da (average: 76326.76 Da), respectively. Most DYW-type PPR genes were located in chloroplasts (91, 70.54%), indicating that this gene family mainly exerts biological functions in the chloroplasts of pears. Other genes were located in the cytoplasm (21), mitochondria (10), nucleus (5), vacuole (1) and plasma membrane (1).

### 3.3 Characterization of gene structure, conserved domains and promoter motifs

**Figure 3:**
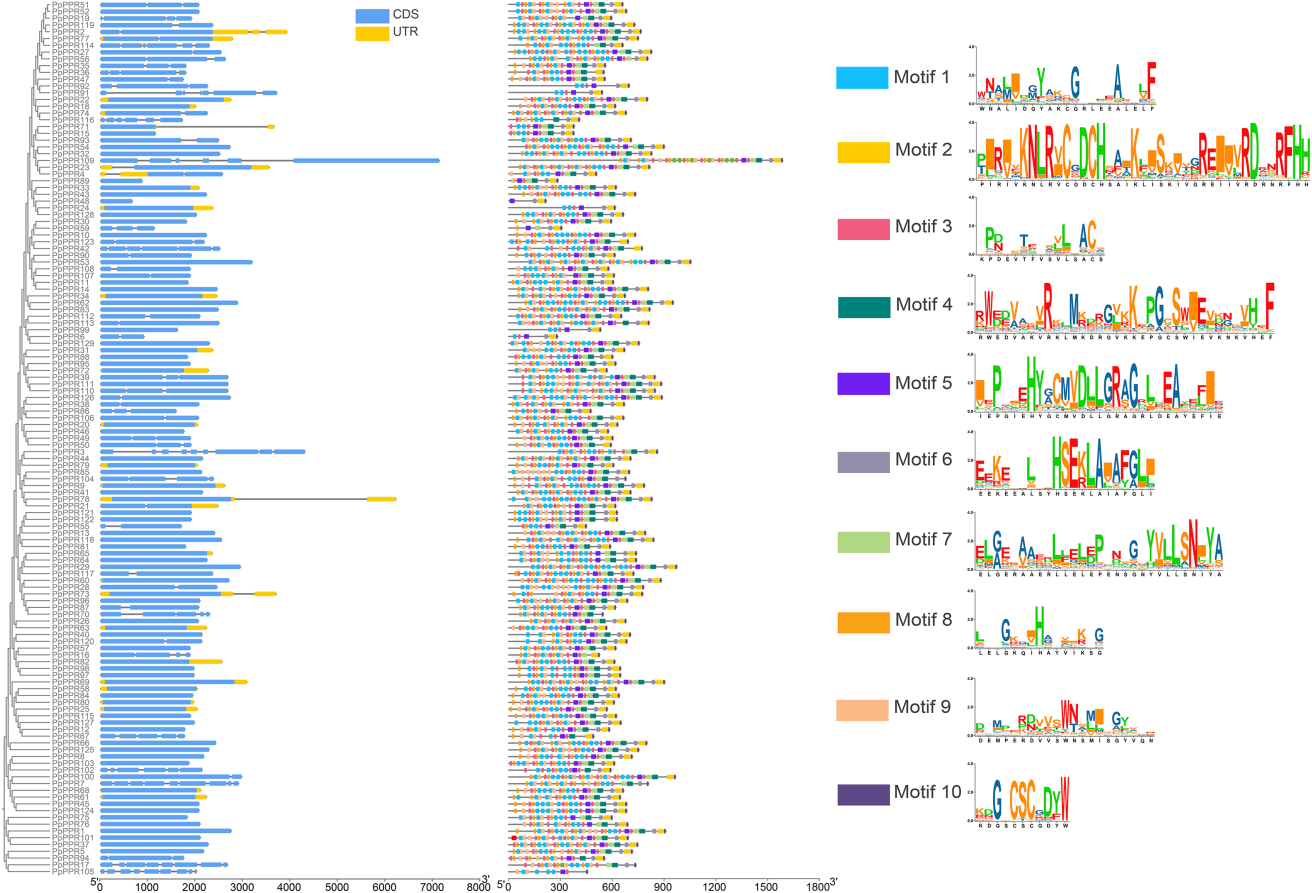
Gene structure of DYW-type PPR gene family and corresponding protein domains. (Left) Gene structures of DYW-type PPR family genes. The grey lines represent introns, and the blocks represent exons. The CDS and UTR of exons are distinguished by blue and yellow, respectively. (Middle) The distribution of ten typical protein domain motifs with different colors on the DYW-type PPR gene sequences. (Right) The amino acid sequence models corresponding to ten typical protein domain motifs. The block colors are characterized by RGB value: Motif 1 (16, 191, 252); Motif 2 (252,204,0); Motif 3 (240,92,128); Motif 4 (0,130,124); Motif 5 (113, 30, 244); Motif 6 (147,143,170); Motif 7 (176,216,130); Motif 8 (251,161,23); Motif 9 (252,184,135); Motif 10 (94,72,134).

**Figure 4:**
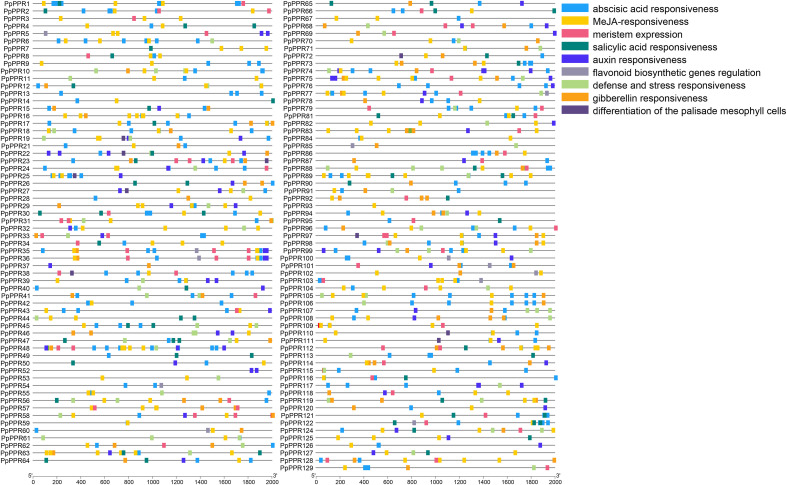
Promoter motifs in DYW-type PPR genes. The straight line represents a 2-kb promoter of DYW-type PPR genes, on which the blocks of various colors represent different types of promoter motifs. The numbers in parentheses represent the quantity of this motif that has been retrieved across all promoters. The block colors are characterized by RGB value: abscisic acid responsiveness (30, 161, 249); MeJA-responsiveness (252, 204, 0); meristem expression (240, 92, 128); salicylic acid responsiveness (0, 130, 124); auxin responsiveness (75, 47, 242); flavonoid biosynthetic genes regulation (147, 143, 170); defense and stress responsiveness (176, 216, 130); gibberellin responsiveness (251, 161, 23); differentiation of the palisade mesophyll cells (94, 72, 134).

All 129 DYW-type PPR genes were subjected to structure classification and identification (**Figure 3**). A total of 27 members contained typical untranslated regions (UTRs). Most members had no or short introns, while a few members, such as *PpPPR71* and *PpPPR78*, harbored long introns. The average length of coding sequences (CDSs) was 2044.17 bp, with maximum and minimum being 4764 and 657 bp, respectively. To investigate the homogeneity of protein sequence within the DYW-type PPR family, the MEME tool was used to predict the top ten conserved domains ordered by E-values (**Figure 3**). Motif 10, comprising 11 amino acids and containing the ‘DYW’ conserved domain, was detected across all family members. Adjacent motifs, motifs 8 and 9, exhibited coverage rates of 96.12% and 93.02%, respectively, underscoring their role as core sequences of the DYW-type PPR family. The diverse conserved sequences were not classified as complete domains due to their short length; however, they are necessary for the DYW-type PPR genes to accomplish their functions. These conserved sequences, composed of 3–5 amino acid residues, were the main structural feature of the DYW-type PPR proteins (**Supplementary Figure S2**). To identify the potential regulatory factors, a total of 129 promoter sequences derived from the DYW-type PPR genes were extracted and subjected to functional motif scanning based on sequence conservation (**Figure 4**). Based on the weighting of transcription factor binding preferences, nine functional motifs distributed across distinct promoter regions were retrieved, representing the diverse transcriptional regulatory landscape of DYW-type PPR proteins. Notably, motifs related to abscisic acid (ABA) and methyl jasmonate (MeJA) signalings pathways were overwhelmingly enriched in 93.8% of the DYW-type PPR members, with 261 and 222 occurrences, respectively. This finding suggested that ABA and jasmonic acid (JA) play critical roles as upstream regulators of DYW-type PPR gene expression.

### 3.4 Intraspecific collinearity and evolutionary analyses

**Figure 5:**
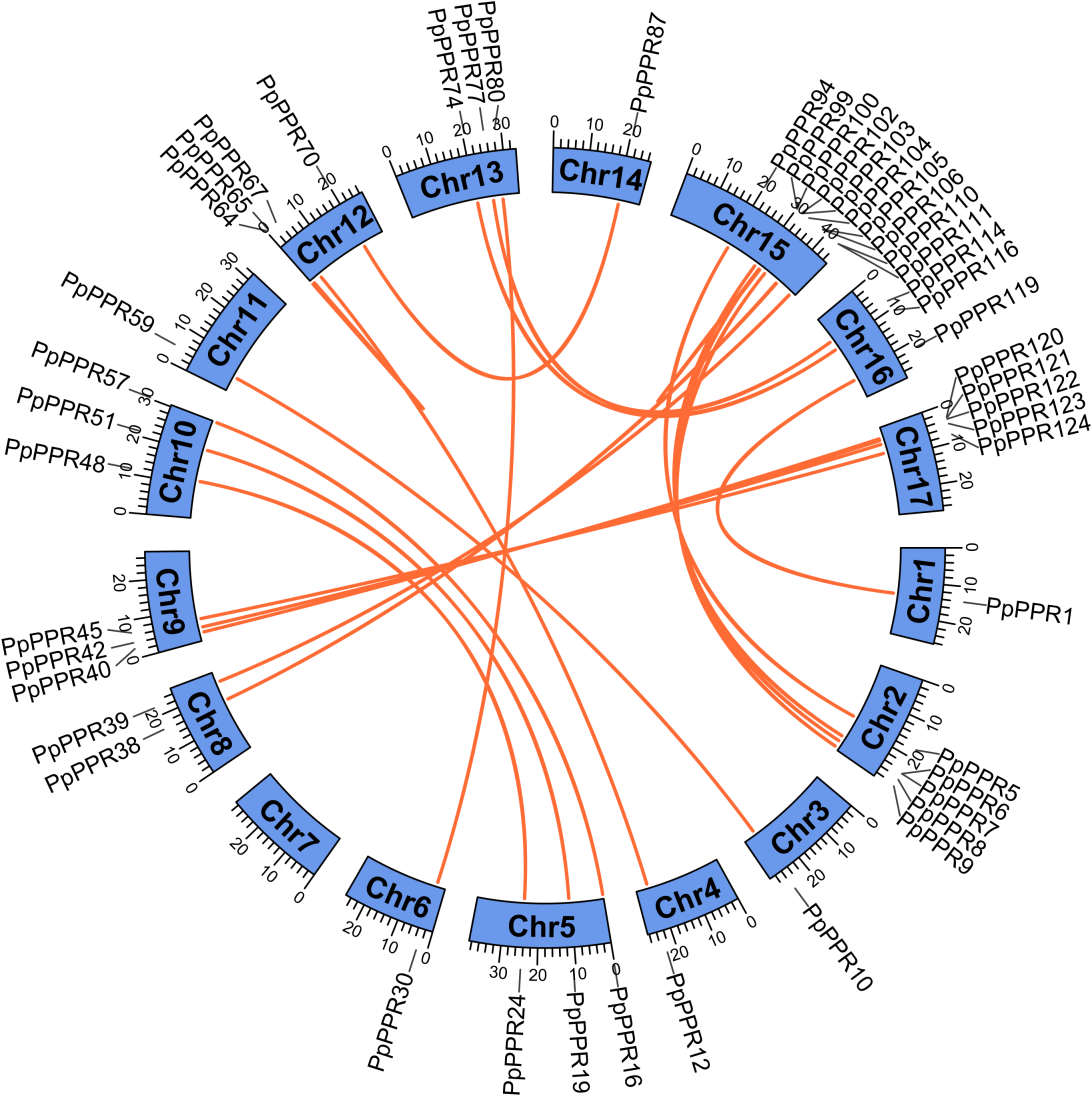
Collinearity analysis of DYW-type PPR genes across different chromosomes. The blue modules represent different chromosomes 1 to 17, with the numbers indicating the base length in kb. The orange line shows the collinear relationship between two DYW-type PPR genes.

We conducted intraspecific collinearity analyses and found 25 collinear events involving 47 DYW-type PPR genes (**Figure 5**). These collinear events are unevenly distributed across 16 chromosomes, except for chr7. Among them, 92% were interchromosomal collinear events. Intrachromosomal collinearity was observed on chr 12 (*PpPPR64* and *PpPPR65*), chr 15 (*PpPPR102* and *PpPPR103*) and chr 17 (*PpPPR121* and *PpPPR122*). These collinear events are associated with gene duplication and contribute to the expansion of the DYW-type PPR gene family. Remarkably, clusters of multiple genes are present on chr2, chr15, and chr17, indicating tandem traces of gene replication and functional co-evolution. In addition, selection pressure analysis was performed to characterize the evolutionary state of intraspecific collinear events (**Supplementary Table S2**). For this purpose, we calculated the Ka/Ks ratio, representing the frequency of non-synonymous substitutions relative to synonymous substitutions. A total of 24 collinear gene pairs were obtained with corresponding Ka/Ks values ranging from 0.12 to 0.64, indicating the absence of positive selection signals and a tendency to undergo purifying selection.

### 3.5 Structural variation analysis of DYW-type PPR genes associated with chlorotic leaves in pears

To identify the structural variation in DYW-type PPR genes associated with pear chlorotic leaves, we conducted high-depth resequencing (50x) using the *P. pyrifolia* (‘Cuiguan’) genome as a reference. A total of 14,402 single nucleotide polymorphisms (SNPs) and 2196 insertions/deletions (InDels) were identified (**Supplementary Figure S3**). These variants were distributed across various gene structures, including intergenic regions, untranslated regions (UTRs), introns, and exons. According to their different characteristics and functional changes, variations in exons were further classified into synonymous mutations, non-synonymous mutations, and frameshift insertions and deletions. Given that CM originates from natural bud mutation, variation sites consistent between ‘Cuiguan’ and CL but differing from CM are more likely to represent internal factors that induce chlorotic leaves and thus possess potential biological significance. Based on this criterion, 33 SNPs and four InDels with potential biological significance were identified, and these were distributed across nine DYW-type PPR genes (**Supplementary Table S3**). Among them, the exon region of *PpPPR75* harbored nine SNPs and two InDel variants, while *PpPPR92* contained seven SNPs and two InDel variants. These two genes exhibited more intense structural variations, which might have changed their biological functions. However, all mutations within the DYW-type PPR exon region are heterozygous, and no homozygous mutations have been identified. These data provided novel insights into the correlation between functional deficits caused by structural variations in DYW-type PPR genes and pear chlorotic leaves.

### 3.6 Expression patterns of DYW-type PPR genes and interaction network of corresponding proteins

**Figure 6:**
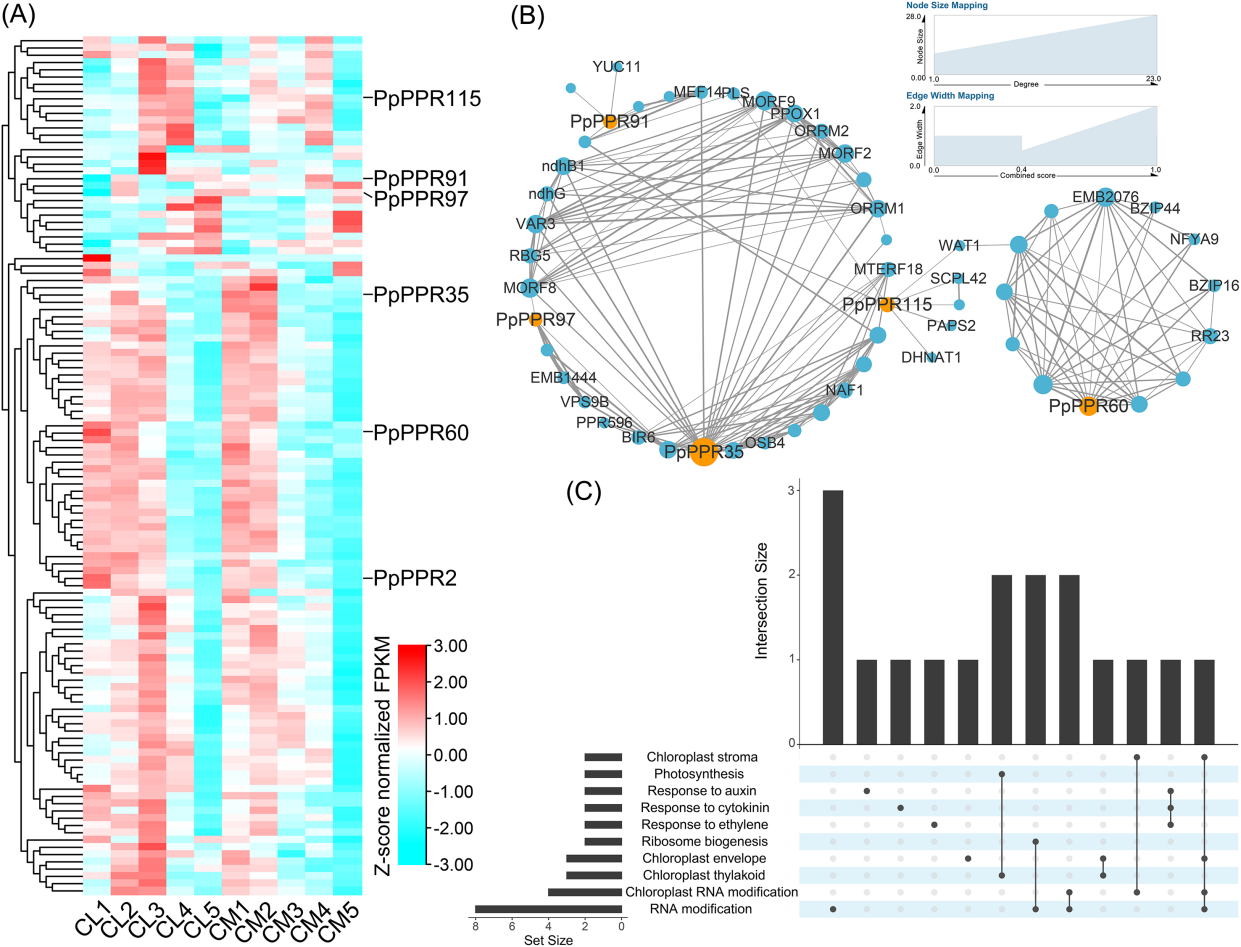
Expression pattern, protein-protein interaction network and functional enrichment of DYW-type PPR genes expressed in pear leaves. **(A)** Expression pattern of DYW-type PPR genes across five stages between CL and CM. The leaf expansion stage was defined as 0 days, and 6, 12, 18, 24 and 30 days were defined as stage 1, stage 2, stage 3, stage 4 and stage 5, respectively. CL1–5 represent stage 1–5 of CL, respectively. CM1–5 represent stage 1–5 of CM, respectively. Z-score normalization was employed to row scale. **(B)** Protein-protein interaction network of DYW-type PPR proteins. The orange node represents DYW-type PPR proteins, and the blue node represents their interacting proteins. The node size reflects the degree number, and the edge width reflects the combined score between two nodes. **(C)** Gene ontology (GO) enrichment of genes in the interaction network. The lower left corner represents different GO terms, and ‘Set size’ represents the number of genes contained in the corresponding GO terms. The X-axis represents different intersection groups composed of one or more GO terms, and the Y-axis represents the number of genes corresponding to different intersection groups. A single black dot in the white and light blue striped area indicates that a gene is included in the corresponding GO term. Two or more black dots connected by a straight line represent that a gene is included in two or more GO terms, respectively.

Transcriptome data from dynamic developmental stages in the yellow-green process of pear leaves were utilized to characterize the expression profiles of DYW-type PPR gene family. The expression abundance of these 129 genes varied markedly across five developmental stages (**Figure 6A**). Six genes with particular and significant expression in the CM *vs*. CL comparison group were identified based on global expression pattern analysis and stage-specific expression differences. Among these, *PpPPR2*, *PpPPR60*, and *PpPPR115* were downregulated in CM1 (S1), CM2 (S2) and CM3 (S3) compared to CL1 (S1), CL2 (S2) and CL3 (S3), respectively (**Figure 6A**). In particular, the expression of *PpPPR115* was down-regulated by 0.15-fold in CM1 compared to CL1, suggesting its potential involvement in chlorotic leaves and the construction and development of chloroplasts. Conversely, *PpPPR35*, *PpPPR91*, and *PpPPR97* were upregulated by 2.05- to 13.14-fold in CM1 compared to CL1, highlighting their potential negative regulatory effects on early leaf and chloroplast development. These six genes were then subjected to protein network construction, with five of them exhibiting typical interactions with other genes (**Figure 6B**). *PpPPR35* demonstrated potential interactions with 23 genes, including members of the MORF and NDH families, which are involved in chloroplast RNA editing and photosynthetic electron transport chains, respectively. The presence of shared interacting genes indicated redundant biological functions among the PPR family genes. For instance, *ndhB1* and *MEF14* interacted with *PpPPR35*-*PpPPR115* and *PpPPR35*-*PpPPR91* modules, respectively, suggesting their involvement in RNA editing and chloroplast structural development via *ndhB1* and *MEF14*, respectively. Additionally, 12 genes interacting with *PpPPR60* formed a distinct cluster, indicating specific functional associations. GO functional enrichment analysis was employed to elucidate the gene functions within the interaction regulatory network (**Figure 6C**). These genes were predominantly involved in chloroplast development, chloroplast RNA editing, photosynthesis, and plant hormone response. Several biological processes occur concurrently, such as photosynthesis and chloroplast thylakoid-related processes (*ndhG* and *ndhB1*) and ribosome biogenesis and RNA modification (*MORF2* and *NAF1*), suggesting their potential involvement in similar biological functions. Interestingly, two genes involved in auxin response, *POLARIS* (*PLS*) and (*WALLS ARE THIN 1*) *WAT1*, were found to interact exclusively with *PpPPR115* (**Figure 6B**), thereby underscoring the role of *PpPPR115* in the auxin signaling pathway.

### 3.7 Construction of weighted gene co-expression network

**Figure 7:**
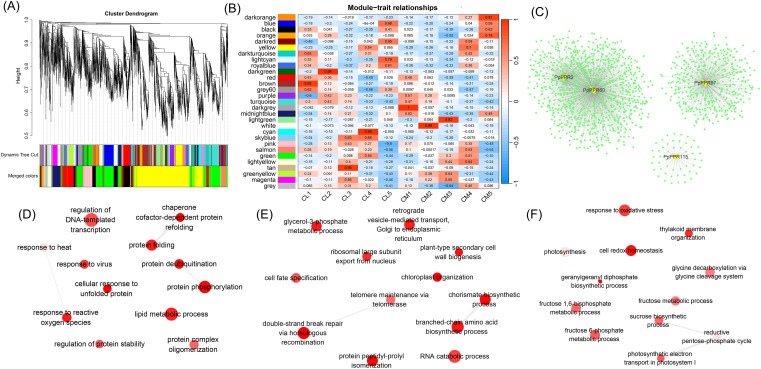
Module identification and enrichment analysis of DYW-type PPR genes based on weighted gene co-expression network analysis (WGCNA). **(A)** Tree diagram showing WGCNA. **(B)** Relationships of coexpression modules and different development stages. The leaf expansion stage was defined as 0 days, and 6, 12, 18, 24 and 30 days were defined as stage 1, stage 2, stage 3, stage 4 and stage 5, respectively. CL1–5 represent stage 1–5 of CL, respectively. CM1–5 represent stage 1–5 of CM, respectively. **(C)** Co-expression network of four DYW-type PPR genes with differential expression. The GO enrichment of genes stems from gene cluster of *PpPPR2* and *PpPPR60***(D)**, *PpPPR97***(E)**, and *PpPPR115***(F)** respectively in the co-expression network. A bubble represents an individual GO term. Bubble color indicates the *P*-value, and dark red represents a high *P*-value. Bubble size indicates the frequency of the GO term in the underlying GOA database. Highly similar GO terms are linked by a grey line, and the grey line width indicates the degree of similarity.

To further investigate the co-expression pattern of the DYW-type PPR genes during pear chlorotic leaves, a total of 31237 genes were subjected to WGCNA, and a tree diagram containing different modules was constructed (**Figure 7A**). A total of 28 specific expression modules (excluding the grey module) were identified, with 117 DYW-type PPR genes distributed across 12 of these modules (**Figure 7B**; **Supplementary Figure S4**). Some modules display distinct expression patterns. For instance, in the first three stages, CL1, CL2 and CL3 in the black module are more highly expressed than CM1, CM2 and CM3, respectively, which is similar to the change in the yellowing and greening state of CM leaves. Therefore, the genes in this module may positively regulate chloroplast development and chlorophyll synthesis, and act as potential regulatory factors responsible for the early yellowing (first three stages) of CM leaves (**Figure 1A**). The gene set in the blue module is predominantly upregulated in the final stage and may be associated with leaf maturation and senescence. For the gene set in the brown module, CL expression reaches its peak during the initial stage and declines thereafter, suggesting that these genes may play a crucial role in early leaf development, including chloroplast structural organization and morphogenesis. Additionally, we performed a correlation analysis between gene modules and developmental stages (**Figure 7B**). The brown, darkgreen, tan, cyan, darkgrey, white and lightgreen modules show strong correlations with CL1 (0.92), CL2 (0.99), CL3 (0.95), CL4 (0.99), CM1 (1), CM2 (0.99) and CM3 (0.93) respectively, indicating that these gene modules have specific roles at different stages of leaf development.

Building upon the prior differential expression and module identification analyses, we constructed a co-expression network centered around four key genes (**Figure 7C**). These genes and their co-expressed genes formed three clusters and were subject to GO enrichment analysis. The first 12 GO terms (biological process) sorted by *P*-value are shown in bubbles. *PpPPR2*, *PpPPR60* and *PpPPR97* originated from the brown module. The co-expression network shared by *PpPPR2* and *PpPPR60* comprised 997 genes, which were primarily enriched in 12 GO terms including DNA-templated transcription regulation, lipid metabolic and protein phosphorylation (**Figure 7D**). *PpPPR97* forms an independent co-expression network cluster, with associated genes mainly involved in 12 GO terms including RNA catabolism and plant-type secondary cell wall formation (**Figure 7E**). However, *PpPPR115* come from the black module. The co-expression network centered on *PpPPR115* is relatively small, encompassing only 51 genes (**Figure 7F**), yet it is significantly enriched in biological processes related to early chloroplast development, including photosynthesis, photosynthetic electron transport in photosystem I and thylakoid membrane organization. These findings underscore the key role of *PpPPR115* in the greening of the chlorotic leaves in pear.

### 3.8 Analysis of transcriptional regulation of *PpPPR115*

**Figure 8:**
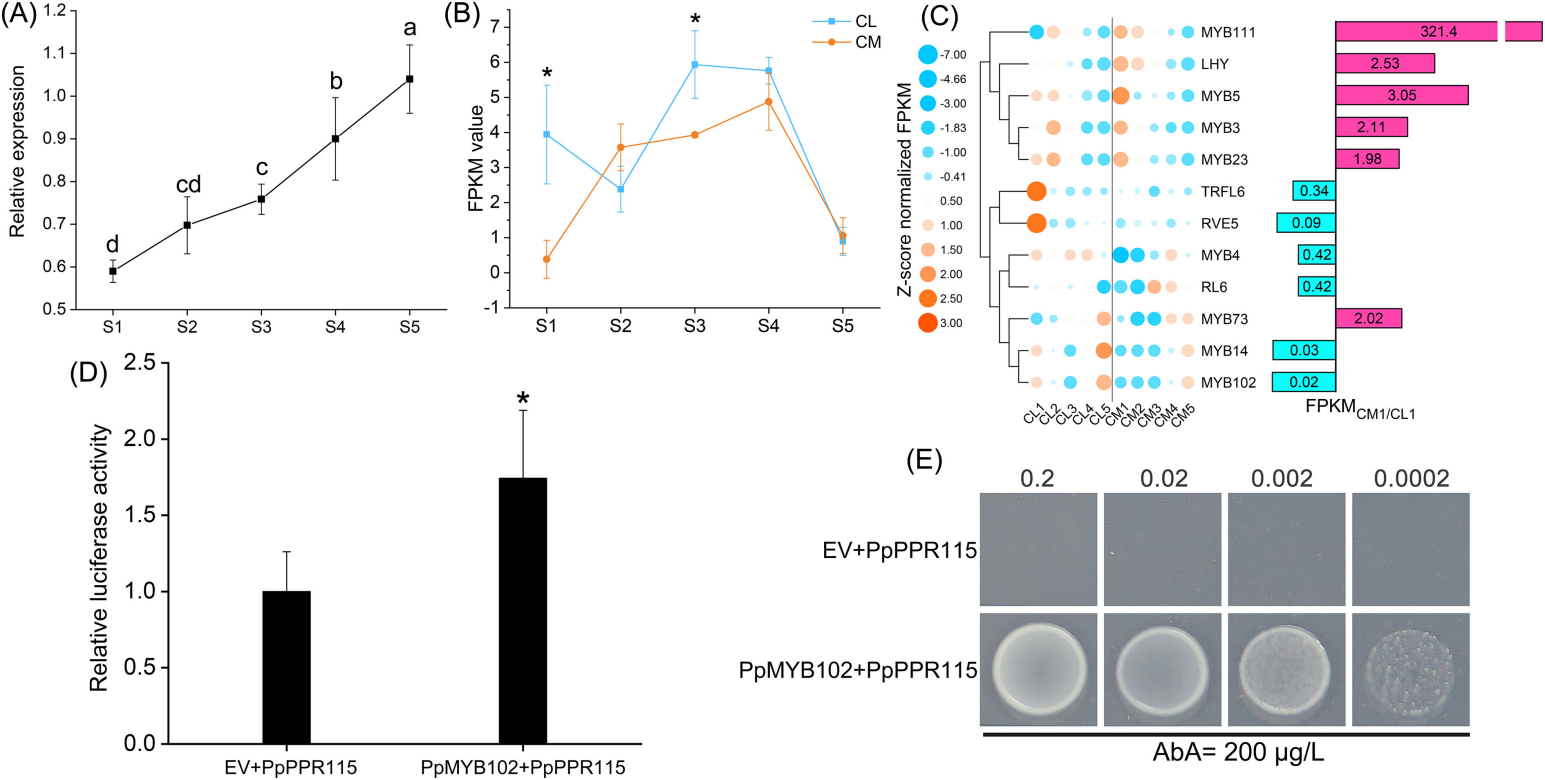
The expression pattern and transcriptional regulation analysis of *PpPPR115*. **(A)** The relative expression of *PpPPR115* in CM *vs*. CL across five developmental stages. Statistical significance was determined by Fisher’s LSD test and Duncan test (*P < 0.05). Three replicates are applied to each sample. **(B)** The expression pattern of *PpPPR115* across five developmental stages in the CL and CM comparison group. Statistical significance was determined by an independent samples t-test (*P < 0.05). **(C)** The expression tendency and expression ratio of MYB family genes across five developmental stages in the CL and CM comparison group. The pink bar indicates the upregulated genes in CM *vs*. CL. The blue bar indicates the downregulated genes in CM *vs*. CL. Z-score normalization was employed to row scale. **(D)** The dual-luciferase assay of the bait plasmid *PpPPR115* activated by the prey plasmid *PpMYB102*. Statistical significance was determined by an independent samples t-test (*P < 0.05). **(E)** The yeast one-hybrid status of the bait plasmid *PpPPR115* activated by the prey plasmid *PpMYB102*. The number at the top represents the OD_600_ value of the yeast strain. AbA, Aureobasidin A.

Previous analyses suggested a close association between *PpPPR115* and pear chlorotic leaves. To further elucidate this coupling relationship, quantitative polymerase chain reaction (PCR) was utilized to assess the dynamic expression profiles of *PpPPR115*. The relative expression levels of this gene during the first three developmental stages were 0.59, 0.70, and 0.76, respectively; however, no significant differences were observed in its relative expression levels during the last two developmental stages (**Figure 8A**). Transcriptome-based expression trends of *PpPPR115* also revealed fluctuations during the first three developmental stages, especially in the S1 stage, where the expression of CM was downregulated by 0.147-fold compared to control (CL, **Figure 8B**). This result aligned with the dynamic changes in leaf color appearance and morphology.

While uncovering the upstream regulatory mechanism of *PpPPR115*, two potential MYB family binding sites were identified in the *PpPPR115* promoter sequence, located at positions 1086 bp and 1098 bp upstream of the transcriptional start site. Based on this finding, the expression patterns of 12 MYB genes with significant expression differences were characterized (**Figure 8C**). Six MYB genes were upregulated during the yellowing stages (CM1 *vs*. CL1), with *PpMYB111* showing a 321.4-fold increase in expression, significantly higher than other MYB genes, indicating its potential as a dominant negative regulator in the early stage of leaf development. Conversely, the downregulated MYB genes might positively regulate *PpPPR115*, promoting leaf and chloroplast development. Furthermore, we cloned the sequences of the *PpPPR115* promoter and MYB genes and conducted dual-luciferase assays, which revealed that *PpMYB102* positively regulated *PpPPR115* (**Figure 8D**). In addition, yeast one-hybrid assays demonstrated that the bait vector containing the *PpPPR115* promoter fragment was unable to support the growth of yeast strains on selective medium supplemented with aureobasidin A (AbA) following co-transformation with an empty prey vector. However, co-transformation with the prey plasmid expressing the full CDS of *PpMYB102* conferred AbA resistance to the yeast strain. These results indicated the interaction between the *PpPPR115* promoter fragment containing the MYB binding site and *PpMYB102* (**Figure 8E**).

## 4 Discussion

Genome-wide analysis provides an overview of the structural and functional annotations of individual family genes and is widely used in the study of multiple species and traits (Dai et al., 2023; Lu et al., 2025). Pear is a widely eaten fruit in the world and exhibits excellent market competitiveness in terms of both fresh consumption and processing. However, chlorotic leaves have emerged as a critical issue limiting the development of the pear industry. The primary internal cause of chlorotic leaves is abnormal chloroplast development, which is mediated by the synergistic effects of multiple factors. Since chloroplasts possess independent genomes, RNA transcription generates primary transcripts containing introns and untranslated terminal sequences. These transcripts require post-transcriptional modifications mediated by RNA splicing complexes centered on PPR proteins (Delannoy et al., 2007). DYW-type PPR proteins, characterized by a unique DYW domain, play a master role in RNA editing, particularly C-to-U editing (Maeda et al., 2022). A previous study has shown that the knockout of the rice DYW subfamily gene *OsPPR16* resulted in reduced accumulation of rpoB protein, a core subunit of PEP, and downregulation of PEP-dependent genes, resulting in an abnormal chloroplast (Huang et al., 2020). The insufficient activity of unedited rpoB cannot support rapid chloroplast division and development, a process predominantly regulated by DYW-type PPR proteins (Zhou et al., 2009). The DYW domain confers cytidine deaminase activity and is highly conserved for RNA editing (Hayes et al., 2015; Oldenkott et al., 2019). Nevertheless, several studies have suggested that certain PPR proteins lacking the DYW domain can trans-recruit the DYW domain to acquire RNA editing activity (Guillaumot et al., 2017; Oldenkott et al., 2020). Among the 129 DYW-type PPR proteins identified in pear, some proteins do not harbor a completely conserved ‘DYW’ domain (**Figure 3**), yet their highly homologous amino acid sequences classify them into the DYW subfamily. These members might possess DYW domain recruitment activity to compensate for their non-standard “DYW” amino acid sequences. In addition to the typical DYW domain, the PG box is also an essential structure for PPR proteins to exert their editing activity. Single- or multi-base mutations in flanking sequences can significantly reduce the editing efficiency of these proteins, potentially explaining the varying editing activities among DYW-type PPR family members (Toma-Fukai et al., 2023; Wang and Takenaka, 2025). In the present study, we identified ten representative domain motifs within the DYW-type PPR family. Among these, the PG box with different flanking sequences is located in motif 4. Base differences near the PG box will be investigated in our subsequent research to assess their impact on the editing efficiency of proteins.

The MORF family proteins, also known as RNA editing factor-interacting proteins, form an editosome with PPR proteins and organelle RNA recognition motif family members (Andrew B et al., 2021). *In vitro* synthesis techniques have been utilized to study the functional roles of PPR proteins. Artificially synthesized P-type PPR proteins with DYW domains exhibit RNA editing effects similar to DYW-type PPR proteins but only when MORF family members are present (Sébastien et al., 2025). Heat stress in Arabidopsis inhibits MORF8-mediated RNA editing for NADH dehydrogenase (NDH/ndh), affecting chloroplast development (Wu et al., 2025). This finding indicated that PPR proteins, MORF family members, and NDH can form a functional complex under specific conditions. Moreover, the function of the DYW-type PPR protein CREF7 in editing the *ndhB* gene has been elucidated (Hayes et al., 2015). This gene mainly encodes the NADH dehydrogenase subunit involved in the establishment of the photosynthetic electron transfer chain, and in this study, it corresponds to *PpPPR115*. In the current study, six DYW-type PPR proteins were differentially expressed between normal and pear chlorotic leaves, with potential interactions with three MORF proteins and two NDHs (**Figure 6A, B**), indicating that PPR proteins, MORF and NDH might work synergistically to regulate the photosynthetic electron transfer chain during the chloroplast development process. Furthermore, functional enrichment analysis revealed that some genes within the PPR protein interaction network might be regulated by plant hormones (**Figure 6C**). Alternatively, several plant hormone-responsive genes interact with DYW-type PPR proteins to mediate their RNA editing activity. Previous studies have demonstrated that *AtYUC2* regulates the expression of several RNA editing factors and participates in the RNA editing of the *ndhD*-2 and *rps14–*149 loci, a process dependent on the expression of *AtARF1* (Li et al., 2023). In the protein interaction network (**Figure 6B**), *PLS* and *WAT1*—proteins associated with auxin signaling—interact specifically with *PpPPR115*, highlighting a potential functional link among *PpPPR115*, auxin signaling, and chlorotic leaf phenotypes. However, this observation does not exclude the possibility that other DYW-type PPR proteins may also interact with components of the auxin pathway. The complex regulatory interplay between PPR proteins and auxin signaling remains to be fully elucidated, though targeted investigations hold promise for uncovering such mechanisms. Not all PPR proteins serve as primary regulators of chloroplast development and photosynthesis. Co-expression network combined with functional enrichment analysis indicated that *PpPPR2*, *PpPPR60*, and *PpPPR97* were not significantly enriched in terms associated with chloroplast development (**Figure 7D-E**). Interestingly, although the co-expressed genes of *PpPPR115* are fewer than those corresponding to the other three genes, they were significantly enriched in photosynthesis and photosynthetic electron transport in photosystem I (**Figure 7F**), highlighting the master role of *PpPPR115* and its co-expressed genes in chloroplast development and photosynthesis across the leaf development of pear. RNA editing mediated by DYW-type PPR will be given priority in the following work, which was not developed and advanced in this study. The key task of this study was to identify six DYW-type PPR genes with differential expression patterns (**Figure 6A**). Among these, *PpPPR2* was present in the co-expression network derived from WGCNA but absent from the PPI network (**Figure 6B**, **7C**), suggesting that it might function alone. *PpPPR35* and *PpPPR91* exhibited opposite characteristics, indicating that they might perform their functions in polymeric forms. Multi-dimensional analyses revealed the distinct roles of DYW-type PPR family members. Although the expression profiles of *PpMYB102* and *PpPPR115* were not consistent, yeast-one hybrid assays and dual-luciferase experiments demonstrated a robust interaction between them, implying that additional factors beyond *PpMYB102* may regulate *PpPPR115* transcription. Future studies should further explore this regulatory mechanism to enhance our understanding of *PpPPR115* transcriptional regulation.

In conclusion, 129 DYW-type PPR proteins were identified in the *P. pyrifolia* genome, and their evolutionary relationships, protein domains, and promoter motifs were systematically characterized. Based on the analysis of expression patterns, protein interactions and co-expression networks, several key DYW-type PPR proteins potentially related to pear chlorotic leaves were identified. *PpPPR115* was found to be involved in chloroplast development and transcriptionally regulated by *PpMYB102*. These findings provided novel insights into the functions of DYW-type PPR proteins and the *PpMYB102*-*PpPPR115* module in chlorotic leaves and chloroplast morphogenesis in pear.

## Data Availability

The datasets presented in this study can be found in online repositories. The names of the repository/repositories and accession number(s) can be found in the article/**Supplementary Material**. The raw data of whole-genome resequencing and RNA-seq data is accessible in the NCBI (https://www.ncbi.nlm.nih.gov/), via accession numbers PRJNA1310269 and PRJNA1313356.

## References

[B1] Andrew B G. Maureen R H. Stéphane B. (2021). The RanBP2 zinc finger domains of chloroplast RNA editing factor OZ1 are required for protein-protein interactions and conversion of C to U. Plant J. 109, 215–226. doi: 10.1111/tpj.15569, PMID: 34743362

[B2] Bailey T. Johnson J. Grant C. Noble W. (2015). The MEME suite. Nucleic Acids Res. 43, W39–W49. doi: 10.1093/nar/gkv416, PMID: 25953851 PMC4489269

[B3] Bjellqvist B. Hughes G. Pasquali C. Paquet N. Ravier F. Sanchez J. . (1993). The focusing positions of polypeptides in immobilized pH gradients can be predicted from their amino acid sequences. Electrophoresis 14, 1023–1031. doi: 10.1002/elps.11501401163, PMID: 8125050

[B4] Bu D. Luo H. Huo P. Wang Z. Zhang S. He Z. . (2021). KOBAS-i: intelligent prioritization and exploratory visualization of biological functions for gene enrichment analysis. Nucleic Acids Res. 49, W317–W325. doi: 10.1093/nar/gkab447, PMID: 34086934 PMC8265193

[B5] Cao S. Liu R. Wang M. Sun F. Sayyed A. Shi H. . (2022). The small PPR protein SPR2 interacts with PPR–SMR1 to facilitate the splicing of introns in maize mitochondria. Plant Physiol. 190, 1763–1776. doi: 10.1093/plphys/kiac379, PMID: 35976145 PMC9614438

[B6] Cao Z. Yu Q. Sun Y. Lu Y. Cui Y. Yang Z. (2011). A point mutation in the pentatricopeptide repeat motif of the AtECB2 protein causes delayed chloroplast development. J. Integr. Plant Biol. 53, 258–269. doi: 10.1111/j.1744-7909.2011.01030.x, PMID: 21294841

[B7] Chen C. Chen H. Zhang Y. Thomas H. Frank M. He Y. . (2020). TBtools: An integrative toolkit developed for interactive analyses of big biological data. Mol. Plant 13, 1194–1202. doi: 10.1016/j.molp.2020.06.009, PMID: 32585190

[B8] Cheng S. Gutmann B. Zhong X. Ye Y. Fisher M. F. Bai F. . (2016). Redefining the structural motifs that determine RNA binding and RNA editing by pentatricopeptide repeat proteins in land plants. Plant J. 85, 532–547. doi: 10.1111/tpj.13121, PMID: 26764122

[B9] Chou K. Shen H. (2008). Cell-PLoc: a package of Web servers for predicting subcellular localization of proteins in various organisms. Nat. Protoc. 3, 153–162. doi: 10.1038/nprot.2007.494, PMID: 18274516

[B10] Dai M. Zhou N. Zhang Y. Zhang Y. Ni K. Wu Z. . (2023). Genome-wide analysis of the SBT gene family involved in drought tolerance in cotton. Front. Plant Sci. 13. doi: 10.3389/fpls.2022.1097732, PMID: 36714777 PMC9875013

[B11] Delannoy E. Stanley W. A. Bond C. S. Small I. D. (2007). Pentatricopeptide repeat (PPR) proteins as sequence-specificity factors in post-transcriptional processes in organelles. Biochem. Soc. Trans. 35, 1643–1647. doi: 10.1042/bst0351643, PMID: 18031283

[B12] Gehlenborg, Nils (2015). UpSetR: A more scalable alternative to Venn and Euler diagrams for visualizing intersecting sets. doi: 10.32614/CRAN.package.UpSetR

[B13] Guillaumot D. Lopez-Obando M. Baudry K. Avon A. Rigaill G. Falcon de Longevialle A. . (2017). Two interacting PPR proteins are major Arabidopsis editing factors in plastid and mitochondria. Proc. Natl. Acad. Sci. U.S.A. 114, 8877–8882. doi: 10.1073/pnas.1705780114, PMID: 28760958 PMC5565446

[B14] Gutmann B. Royan S. Schallenberg-Rüdinger M. Lenz H. Castleden I. R. McDowell R. . (2020). The expansion and diversification of pentatricopeptide repeat RNA-editing factors in plants. Mol. Plant 13, 215–230. doi: 10.1016/j.molp.2019.11.002, PMID: 31760160

[B15] Hayes M. L. Dang K. N. Diaz M. F. Mulligan R. M. (2015). A conserved glutamate residue in the C-terminal deaminase domain of pentatricopeptide repeat proteins is required for RNA editing activity. J. Biol. Chem. 290, 10136–10142. doi: 10.1074/jbc.M114.631630, PMID: 25739442 PMC4400329

[B16] He H. Cheng M. Bao B. Tian Y. Zheng Y. Huo Y. . (2025). GhCTEF2 encodes a PLS-type PPR protein required for chloroplast development and plastid RNA editing in cotton. Plant Sci. 355, 112478. doi: 10.1016/j.plantsci.2025.112478, PMID: 40107517

[B17] Huang C. Liu D. Li Z. A. Molloy D. P. Luo Z. F. Su Y. . (2023). The PPR protein RARE1-mediated editing of chloroplast *accD* transcripts is required for fatty acid biosynthesis and heat tolerance in *Arabidopsis*. Plant Commun. 4, 100461. doi: 10.1016/j.xplc.2022.100461, PMID: 36221851 PMC9860180

[B18] Huang C. Yu Q. B. Li Z. R. Ye L. S. Xu L. Yang Z. N. (2017). Porphobilinogen deaminase HEMC interacts with the PPR-protein AtECB2 for chloroplast RNA editing. Plant J. 92, 546–556. doi: 10.1111/tpj.13672, PMID: 28850756

[B19] Huang W. Zhang Y. Shen L. Fang Q. Liu Q. Gong C. . (2020). Accumulation of the RNA polymerase subunit RpoB depends on RNA editing by OsPPR16 and affects chloroplast development during early leaf development in rice. New Phytol. 228, 1401–1416. doi: 10.1111/nph.16769, PMID: 32583432 PMC7689822

[B20] Huo Y. Cheng M. Tang M. Zhang M. Yang X. Zheng Y. . (2024). GhCTSF1, a short PPR protein with a conserved role in chloroplast development and photosynthesis, participates in intron splicing of *rpoC1* and *ycf3–2* transcripts in cotton. Plant Commun. 5, 100858. doi: 10.1016/j.xplc.2024.100858, PMID: 38444162 PMC11211521

[B21] Lan J. Lin Q. Zhou C. Liu X. Miao R. Ma T. . (2023). Young Leaf White Stripe encodes a P-type PPR protein required for chloroplast development. J. Integr. Plant Biol. 65, 1687–1702. doi: 10.1111/jipb.13477, PMID: 36897026

[B22] Langfelder P. Horvath S. (2008). WGCNA: an R package for weighted correlation network analysis. BMC Bioinf. 9, 559. doi: 10.1186/1471-2105-9-559, PMID: 19114008 PMC2631488

[B23] Lescot M. Déhais P. Thijs G. Marchal K. Moreau Y. Van de Peer Y. . (2002). PlantCARE, a database of plant cis-acting regulatory elements and a portal to tools for in *silico* analysis of promoter sequences. Nucleic Acids Res. 30, 325–327. doi: 10.1093/nar/30.1.325, PMID: 11752327 PMC99092

[B24] Letunic I. Khedkar S. Bork P. (2020). SMART: recent updates, new developments and status in 2020. Nucleic Acids Res. 49, D458–D460. doi: 10.1093/nar/gkaa937, PMID: 33104802 PMC7778883

[B25] Li J. Chen S. Zhang Y. Zhao W. Yang J. Fan Y. (2024). A novel PLS-DYW type PPR protein OsASL is essential for chloroplast development in rice. Plant Sci. 345, 112134. doi: 10.1016/j.plantsci.2024.112134, PMID: 38810885

[B26] Liu D. Li Z. A. Li Y. Molloy D. P. Huang C. (2023). The DYW domain of RARE1 plays an indispensable role in regulating accD-C794 RNA editing in *Arabidopsis thaliana*. Plant Sci. 334, 111751. doi: 10.1016/j.plantsci.2023.111751, PMID: 37263527

[B27] Love M. I. Huber W. Anders S. (2014). Moderated estimation of fold change and dispersion for RNA-seq data with DESeq2. Genome Biol. 15, 550. doi: 10.1186/s13059-014-0550-8, PMID: 25516281 PMC4302049

[B28] Lu L. Gao X. Qi Y. Zha Z. Gao Z. Ma N. . (2025). Functional characterisation of WRKY transcription factor CrWRKY48 involved in regulating seed abortion of Ponkan (*Citrus reticulata*). Physiologia Plantarum 177, e70048. doi: 10.1111/ppl.70048, PMID: 39829364

[B29] Lu L. Yang H. Xu Y. Zhang L. Wu J. Yi H. (2023). Laser capture microdissection-based spatiotemporal transcriptomes uncover regulatory networks during seed abortion in seedless Ponkan (*Citrus reticulata*). Plant J. 115, 642–661. doi: 10.1111/tpj.16251, PMID: 37077034

[B30] Lurin C. Andrés C. Aubourg S. Bellaoui M. Bitton F. Bruyère C. . (2004). Genome-wide analysis of Arabidopsis pentatricopeptide repeat proteins reveals their essential role in organelle biogenesis. Plant Cell 16, 2089–2103. doi: 10.1105/tpc.104.022236, PMID: 15269332 PMC519200

[B31] Lv Y. Wang Y. Zhang Q. Chen C. Qian Q. Guo L. (2022). WAL3 encoding a PLS-type PPR protein regulates chloroplast development in rice. Plant Sci. 323, 111382. doi: 10.1016/j.plantsci.2022.111382, PMID: 35850283

[B32] Maeda A. Takenaka S. Wang T. Frink B. Shikanai T. Takenaka M. (2022). DYW deaminase domain has a distinct preference for neighboring nucleotides of the target RNA editing sites. Plant J. 111, 756–767. doi: 10.1111/tpj.15850, PMID: 35652245

[B33] Oldenkott B. Burger M. Hein A.-C. Jörg A. Senkler J. Braun H.-P. . (2020). One C-to-U RNA editing site and two independently evolved editing factors: testing reciprocal complementation with DYW-Type PPR proteins from the moss *Physcomitrium* (*Physcomitrella*) *patens* and the flowering plants *Macadamia integrifolia* and Arabidopsis. Plant Cell 32, 2997–3018. doi: 10.1105/tpc.20.00311, PMID: 32616665 PMC7474288

[B34] Oldenkott B. Yang Y. Lesch E. Knoop V. Schallenberg-Rüdinger M. (2019). Plant-type pentatricopeptide repeat proteins with a DYW domain drive C-to-U RNA editing in *Escherichia coli*. Commun. Biol. 2, 85. doi: 10.1038/s42003-019-0328-3, PMID: 30854477 PMC6397227

[B35] Qi W. Yang Y. Feng X. Zhang M. Song R. (2017). Mitochondrial function and maize kernel development requires dek2, a pentatricopeptide repeat protein involved in nad1 mRNA splicing. Genetics 205, 239–249. doi: 10.1534/genetics.116.196105, PMID: 27815362 PMC5223505

[B36] Sébastien M. Elena L. Shahinez G. Stéfanie G. Mareike S.-R. Kamel H. (2025). *De novo* RNA base editing in plant organelles with engineered synthetic P-type PPR editing factors. Nucleic Acids Res. 53, gkaf279. doi: 10.1093/nar/gkaf279, PMID: 40207624 PMC11983096

[B37] Shannon P. Markiel A. Ozier O. Baliga N. S. Wang J. T. Ramage D. . (2003). Cytoscape: a software environment for integrated models of biomolecular interaction networks. Genome Res. 13, 2498–2504. doi: 10.1101/gr.1239303, PMID: 14597658 PMC403769

[B38] Supek F. Bošnjak M. Škunca N. Šmuc T. (2011). REVIGO summarizes and visualizes long lists of gene ontology terms. PloS One 6, e21800. doi: 10.1371/journal.pone.0021800, PMID: 21789182 PMC3138752

[B39] Szklarczyk D. Kirsch R. Koutrouli M. Nastou K. Mehryary F. Hachilif R. . (2023). The STRING database in 2023: protein-protein association networks and functional enrichment analyses for any sequenced genome of interest. Nucleic Acids Res. 51, D638–D646. doi: 10.1093/nar/gkac1000, PMID: 36370105 PMC9825434

[B40] Tan T. Xu S. Liu J. Ouyang M. Zhang J. (2025). A PPR protein RFCD1 affects chloroplast gene expression and chloroplast development in *Arabidopsis*. Plants 14, 921. doi: 10.3390/plants14060921, PMID: 40265857 PMC11944589

[B41] Toma-Fukai S. Sawada Y. Maeda A. Shimizu H. Shikanai T. Takenaka M. . (2023). Structural insight into the activation of an Arabidopsis organellar C-to-U RNA editing enzyme by active site complementation. Plant Cell 35, 1888–1900. doi: 10.1093/plcell/koac318, PMID: 36342219 PMC10226597

[B42] Vasimuddin M. Misra S. Li H. Aluru S. (2019). Efficient architecture-aware acceleration of BWA-MEM for multicore systems. IEEE Int. Parallel Distributed Process. Symposium (IPDPS), 314–324. doi: 10.1109/IPDPS.2019.00041

[B43] Wagoner J. A. Sun T. Lin L. Hanson M. R. (2015). Cytidine deaminase motifs within the DYW domain of two pentatricopeptide repeat-containing proteins are required for site-specific chloroplast RNA editing. J. Biol. Chem. 290, 2957–2968. doi: 10.1074/jbc.M114.622084, PMID: 25512379 PMC4317000

[B44] Wang X. An Y. Qi Z. Xiao J. (2021). PPR protein Early Chloroplast Development 2 is essential for chloroplast development at the early stage of Arabidopsis development. Plant Sci. 308, 110908. doi: 10.1016/j.plantsci.2021.110908, PMID: 34034865

[B45] Wang J. Chitsaz F. Derbyshire M. K. Gonzales N. R. Gwadz M. Lu S. . (2023). The conserved domain database in 2023. Nucleic Acids Res. 51, D384–D388. doi: 10.1093/nar/gkac1096, PMID: 36477806 PMC9825596

[B46] Wang T. Takenaka M. (2025). The molecular basis and evolution of the organellar RNA editosome by complementary DYW deaminases in seed plants. Plant Physiol. 197, kiaf142. doi: 10.1093/plphys/kiaf142, PMID: 40296642

[B47] Wu J. Wang Y. Chen H. Xu T. Yang W. Fang X. (2025). Solid-like condensation of MORF8 inhibits RNA editing under heat stress in Arabidopsis. Nat. Commun. 16, 2789. doi: 10.1038/s41467-025-58146-1, PMID: 40118828 PMC11928522

[B48] Xu M. Zhang X. Cao J. Liu J. He Y. Guan Q. . (2024). OsPGL3A encodes a DYW-type pentatricopeptide repeat protein involved in chloroplast RNA processing and regulated chloroplast development. Mol. Breed. 44, 29. doi: 10.1007/s11032-024-01468-7, PMID: 38549701 PMC10965880

[B49] Zang J. Zhang T. Zhang Z. Liu J. Chen H. (2024). DEFECTIVE KERNEL 56 functions in mitochondrial RNA editing and maize seed development. Plant Physiol. 194, 1593–1610. doi: 10.1093/plphys/kiad598, PMID: 37956067

[B50] Zhang Y. Tian L. Lu C. (2023). Chloroplast gene expression: Recent advances and perspectives. Plant Commun. 4, 100611. doi: 10.1016/j.xplc.2023.100611, PMID: 37147800 PMC10504595

[B51] Zhang B. Wu Y. Li S. Ren W. Yang L. Zhuang M. . (2024). Chloroplast C-to-U editing, regulated by a PPR protein BoYgl-2, is important for chlorophyll biosynthesis in cabbage. Horticulture Res. 11, uhae006. doi: 10.1093/hr/uhae006, PMID: 38559470 PMC10980974

[B52] Zhou W.-B. Cheng Y. Yap A. Chateigner Boutin A. L. Delannoy E. Hammani K. . (2009). The Arabidopsis gene YS1 encoding a DYW protein is required for editing of *rpoB* transcripts and the rapid development of chloroplasts during early growth. Plant J. 58, 82–96. doi: 10.1111/j.1365-313X.2008.03766.x, PMID: 19054358

