# Supplementary material for: Genome-wide identification and dynamic transcriptome profiling of the DYW-type PPR family across greening of chlorotic leaves in pear (Pyrus pyrifolia)
Source: Front Plant Sci. 2026 Jan 29;17:1767760. doi: 10.3389/fpls.2026.1767760 (PMC12894350; doi:10.3389/fpls.2026.1767760)
Supplement: Supplementary file 1 [file Table1.docx]

**Genome-wide identification and dynamic transcriptome profiling of the DYW-type PPR family across greening of chlorotic leaves in pear (*Pyrus pyrifolia*)**

**Liqing Lu^✝^, Haiqi Zhang^✝^, Zixian Zha, Xueqian Wang, Na Ma, Chunyan Liu, Yiliu Xu, Zhenghui Gao^*^, Yongjie Qi^*^**

Key Laboratory of Horticultural Crop Germplasm Innovation and Utilization (Co-Construction by Ministry and Province), Institute of Horticulture Anhui Academy of Agricultural Sciences, Hefei, 230031, China.

*** Correspondence:**Yongjie Qi: qiyongjie@aaas.org.cn.

Zhenghui Gao: gzh96gao@aaas.org.cn.


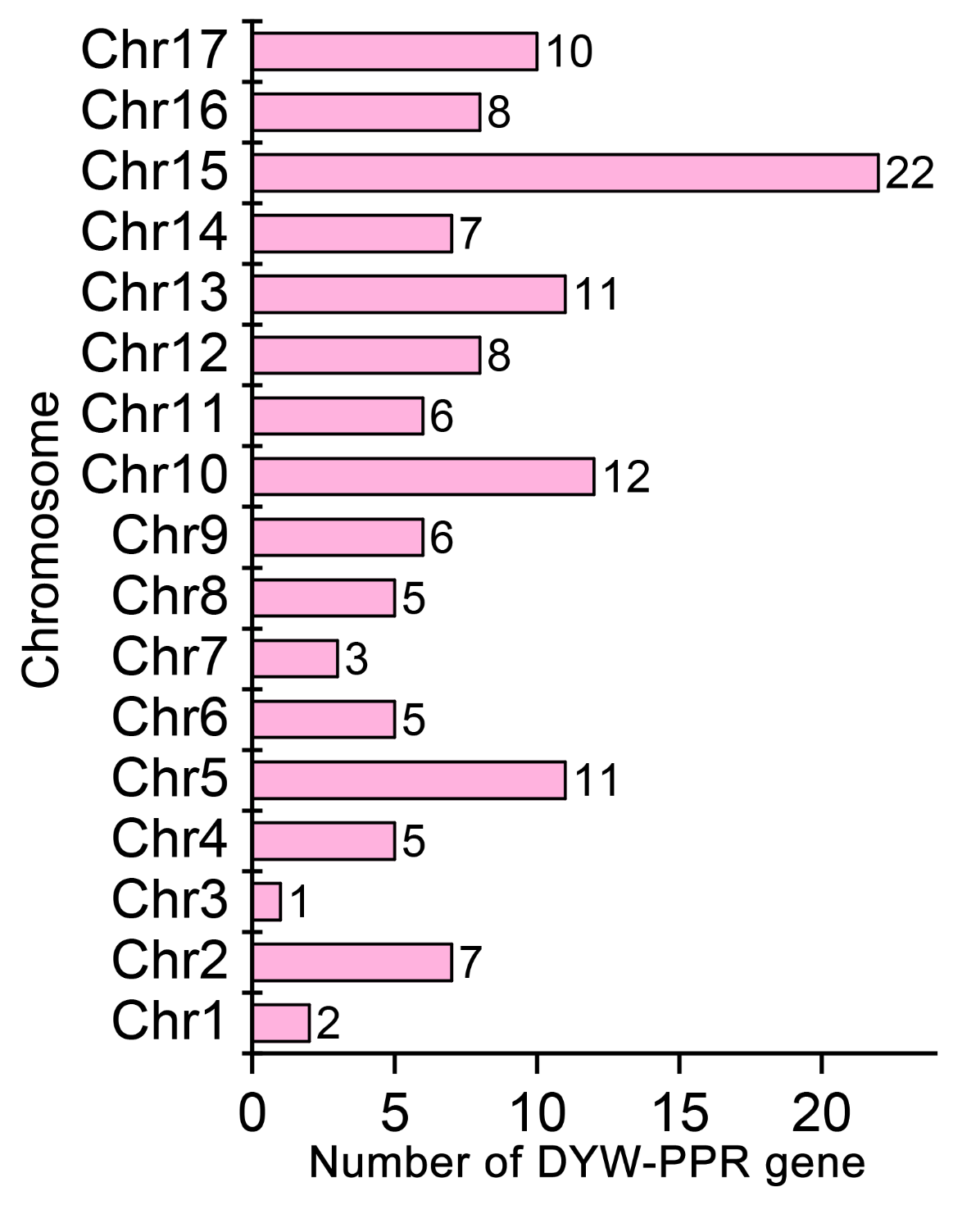


Figure S1 Number of DYW-PPR gene in different chromosomes.


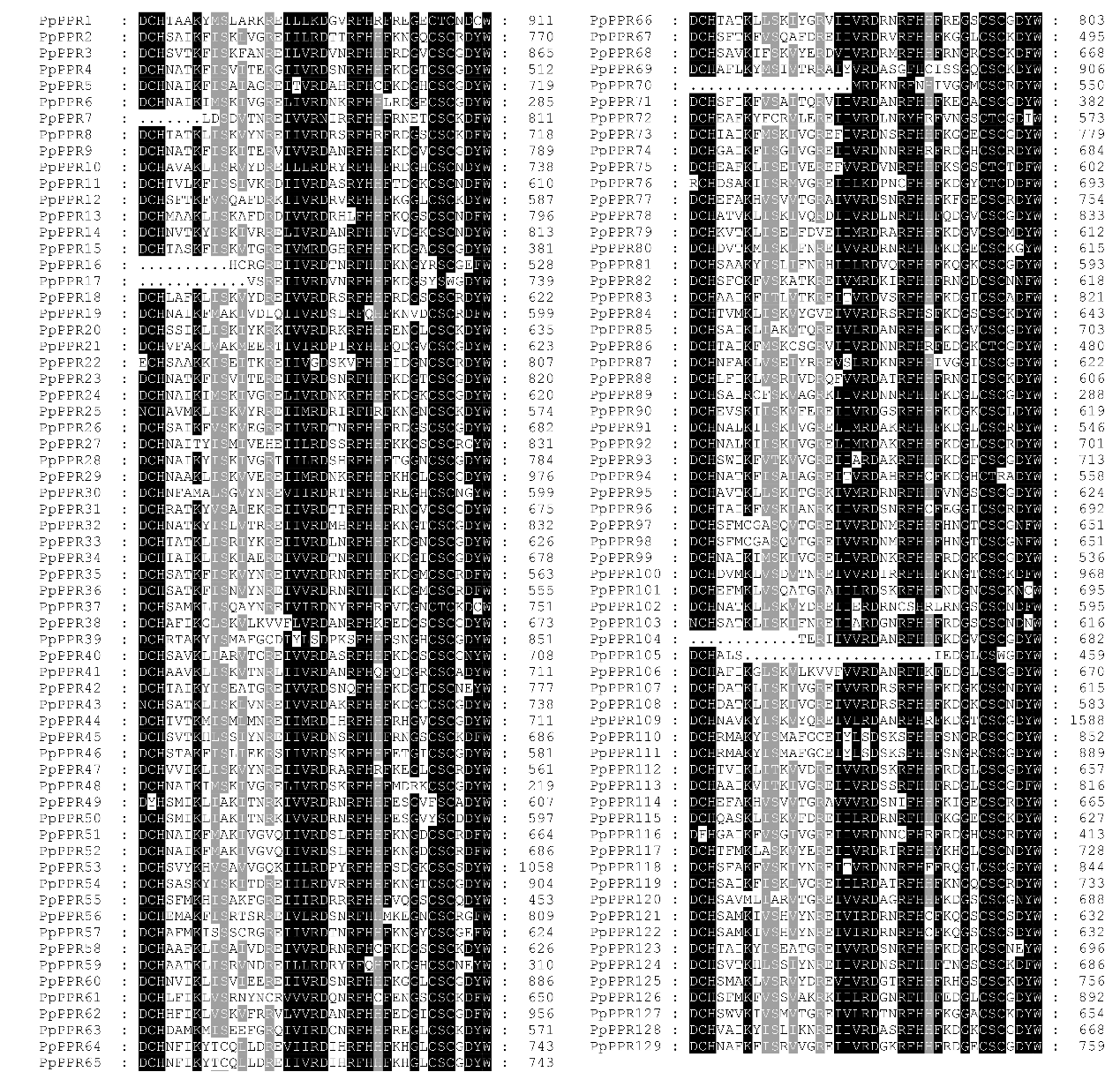


Figure S2 The amino acid sequences of DYW-PPR genes. The black and gray background represent 90% and 70% conservatism, respectively.


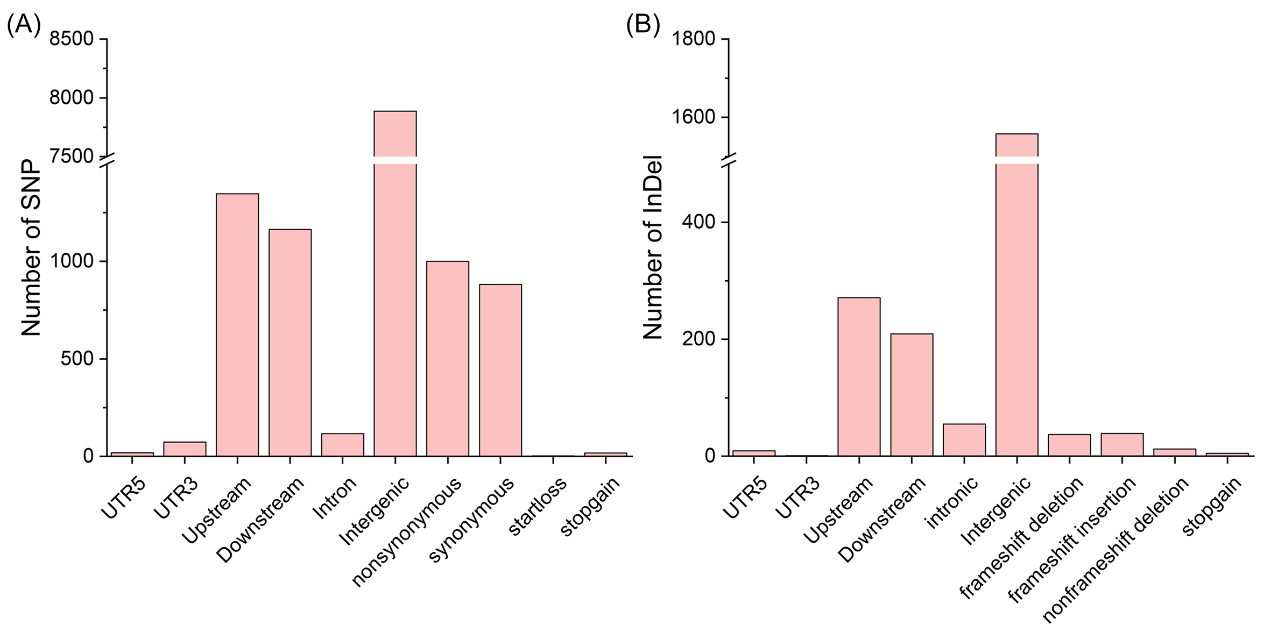


Figure S3 The distribution of SNP (A) and InDel (B) in different gene structures.


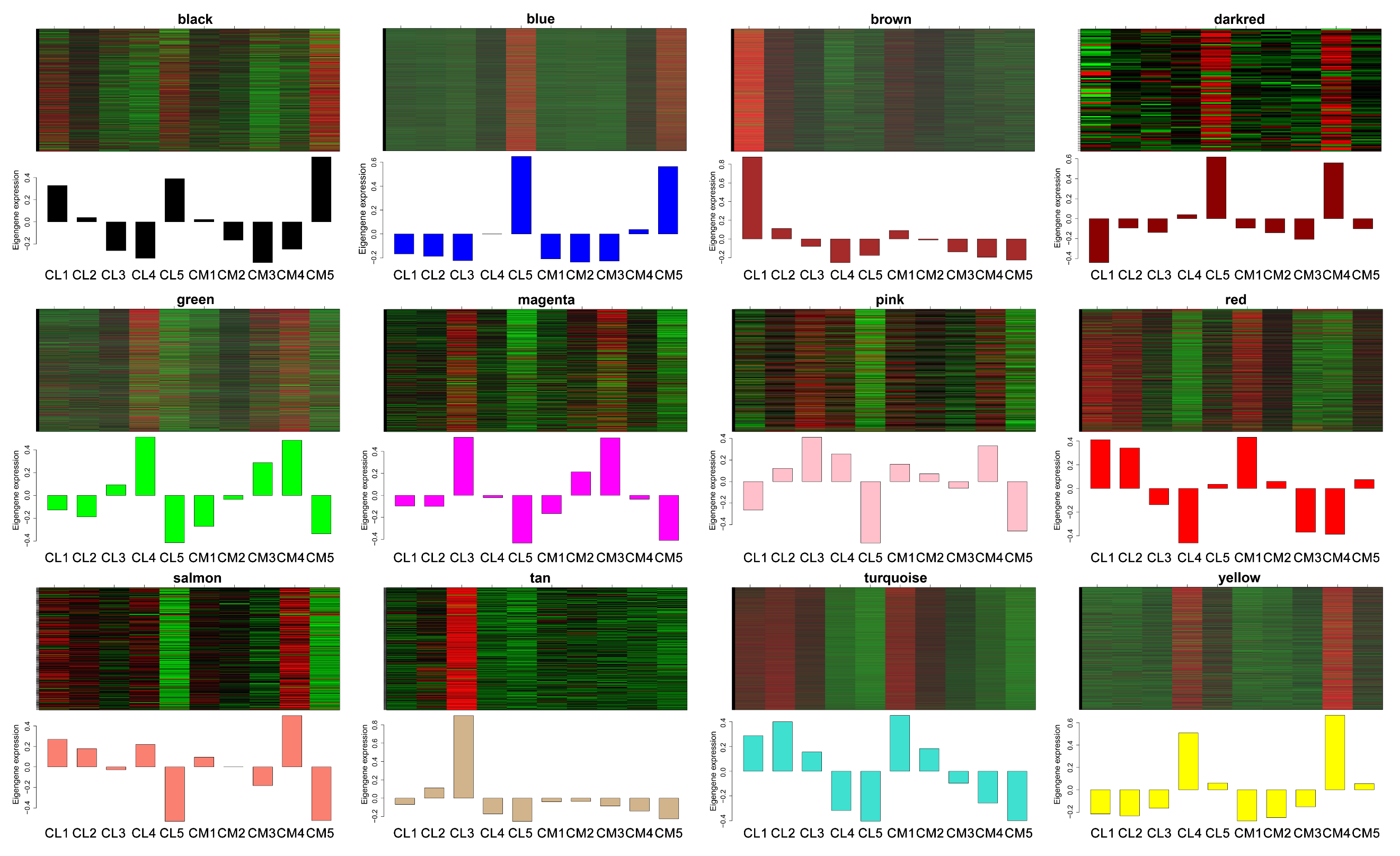


Figure S4 The expressed profiles across 12 samples of 12 key gene modules in WGCNA.
