# Supplementary material for: Genome-wide identification and dynamic transcriptome profiling of the DYW-type PPR family across greening of chlorotic leaves in pear (Pyrus pyrifolia)
Source: Front Plant Sci. 2026 Jan 29;17:1767760. doi: 10.3389/fpls.2026.1767760 (PMC12894350; doi:10.3389/fpls.2026.1767760)
Supplement: Supplementary file 3 [file Table3.docx]

**Genome-wide identification and dynamic transcriptome profiling of the DYW-type PPR family across greening of chlorotic leaves in pear (*Pyrus pyrifolia*)**

**Liqing Lu^✝^, Haiqi Zhang^✝^, Zixian Zha, Xueqian Wang, Na Ma, Chunyan Liu, Yiliu Xu, Zhenghui Gao^*^, Yongjie Qi^*^**

Key Laboratory of Horticultural Crop Germplasm Innovation and Utilization (Co-Construction by Ministry and Province), Institute of Horticulture Anhui Academy of Agricultural Sciences, Hefei, 230031, China.

*** Correspondence:**Yongjie Qi: qiyongjie@aaas.org.cn.

Zhenghui Gao: gzh96gao@aaas.org.cn.

Table S1 Physical and chemical properties of DYW-PPR genes

| gene | pI | mw | sl |  | gene | pI | mw | sl |
| --- | --- | --- | --- | --- | --- | --- | --- | --- |
| PpPPR1 | 7.52 | 103077 | chlo |  | PpPPR66 | 7.84 | 89587 | chlo |
| PpPPR2 | 8.61 | 85695 | cyto |  | PpPPR67 | 8.92 | 55641 | chlo |
| PpPPR3 | 8.53 | 97022 | chlo |  | PpPPR68 | 7.29 | 74673 | nucl |
| PpPPR4 | 5.89 | 57606 | cyto |  | PpPPR69 | 7.06 | 101171 | chlo |
| PpPPR5 | 7.18 | 80306 | chlo |  | PpPPR70 | 6.66 | 62254 | chlo |
| PpPPR6 | 5.91 | 32763 | cyto |  | PpPPR71 | 6.94 | 42910 | cyto |
| PpPPR7 | 5.74 | 89520 | cyto |  | PpPPR72 | 7.9 | 63900 | chlo |
| PpPPR8 | 6.5 | 80295 | chlo |  | PpPPR73 | 8.54 | 86784 | chlo |
| PpPPR9 | 7.37 | 87077 | chlo |  | PpPPR74 | 7.22 | 75398 | chlo |
| PpPPR10 | 6.46 | 82449 | chlo |  | PpPPR75 | 6.87 | 66884 | chlo |
| PpPPR11 | 6.78 | 67928 | chlo |  | PpPPR76 | 8.69 | 78174 | cyto |
| PpPPR12 | 9.02 | 65374 | chlo |  | PpPPR77 | 5.9 | 84682 | chlo |
| PpPPR13 | 6 | 89286 | chlo |  | PpPPR78 | 5.99 | 92656 | chlo |
| PpPPR14 | 8.44 | 91239 | chlo |  | PpPPR79 | 5.96 | 69390 | chlo |
| PpPPR15 | 6.83 | 42894 | chlo |  | PpPPR80 | 7.55 | 69118 | cyto |
| PpPPR16 | 6.99 | 58670 | chlo |  | PpPPR81 | 7.31 | 66438 | chlo |
| PpPPR17 | 8.57 | 83332 | nucl |  | PpPPR82 | 8.95 | 69011 | chlo |
| PpPPR18 | 7.62 | 69828 | chlo |  | PpPPR83 | 8.12 | 91407 | mito |
| PpPPR19 | 7.45 | 67220 | vacu |  | PpPPR84 | 8.55 | 72306 | chlo |
| PpPPR20 | 6.44 | 71189 | chlo |  | PpPPR85 | 6.62 | 79496 | chlo |
| PpPPR21 | 7.22 | 70459 | mito |  | PpPPR86 | 8.43 | 54191 | chlo |
| PpPPR22 | 6.81 | 91311 | chlo |  | PpPPR87 | 6.46 | 70439 | chlo |
| PpPPR23 | 6.79 | 92349 | mito |  | PpPPR88 | 7.23 | 67034 | cyto |
| PpPPR24 | 7.27 | 69937 | mito |  | PpPPR89 | 8.29 | 32695 | chlo |
| PpPPR25 | 8.32 | 64812 | cyto |  | PpPPR90 | 8.17 | 69207 | chlo |
| PpPPR26 | 6.9 | 76852 | mito |  | PpPPR91 | 5.57 | 60343 | cyto |
| PpPPR27 | 7.25 | 92266 | plas |  | PpPPR92 | 5.99 | 79086 | chlo |
| PpPPR28 | 8.47 | 88408 | chlo |  | PpPPR93 | 6.87 | 79423 | chlo |
| PpPPR29 | 6.31 | 107901 | chlo |  | PpPPR94 | 7.56 | 62649 | cyto |
| PpPPR30 | 8.09 | 66930 | chlo |  | PpPPR95 | 8.54 | 70171 | chlo |
| PpPPR31 | 8.55 | 75876 | mito |  | PpPPR96 | 8.29 | 78150 | chlo |
| PpPPR32 | 7.96 | 92820 | chlo |  | PpPPR97 | 6.58 | 72574 | chlo |
| PpPPR33 | 7.52 | 69817 | chlo |  | PpPPR98 | 6.8 | 72662 | chlo |
| PpPPR34 | 6.56 | 75681 | chlo |  | PpPPR99 | 7.57 | 60443 | chlo |
| PpPPR35 | 7.26 | 63069 | cyto |  | PpPPR100 | 5.58 | 106854 | chlo |
| PpPPR36 | 8.17 | 62990 | mito |  | PpPPR101 | 8.31 | 78299 | chlo |
| PpPPR37 | 6.12 | 84604 | cyto |  | PpPPR102 | 5.72 | 67366 | chlo |
| PpPPR38 | 8.14 | 75913 | cyto |  | PpPPR103 | 5.92 | 68260 | cyto |
| PpPPR39 | 8.18 | 95398 | chlo |  | PpPPR104 | 6.32 | 75483 | chlo |
| PpPPR40 | 6.94 | 80392 | nucl |  | PpPPR105 | 9.04 | 52066 | cyto |
| PpPPR41 | 7.54 | 79163 | chlo |  | PpPPR106 | 8.37 | 75320 | cyto |
| PpPPR42 | 5.71 | 86480 | chlo |  | PpPPR107 | 6.85 | 69236 | chlo |
| PpPPR43 | 6.75 | 83142 | chlo |  | PpPPR108 | 7.56 | 65837 | chlo |
| PpPPR44 | 8.05 | 78869 | chlo |  | PpPPR109 | 6.08 | 174574 | chlo |
| PpPPR45 | 6.28 | 77259 | chlo |  | PpPPR110 | 8.36 | 95547 | chlo |
| PpPPR46 | 6.33 | 64898 | chlo |  | PpPPR111 | 8.28 | 99856 | chlo |
| PpPPR47 | 7.49 | 62304 | chlo |  | PpPPR112 | 7.02 | 73451 | chlo |
| PpPPR48 | 7.68 | 24987 | cyto |  | PpPPR113 | 7.78 | 91758 | chlo |
| PpPPR49 | 8.04 | 68808 | chlo |  | PpPPR114 | 7.24 | 74267 | chlo |
| PpPPR50 | 6.71 | 67336 | chlo |  | PpPPR115 | 6.43 | 70300 | chlo |
| PpPPR51 | 8.67 | 74403 | chlo |  | PpPPR116 | 6.24 | 46587 | chlo |
| PpPPR52 | 8.4 | 76657 | chlo |  | PpPPR117 | 6.07 | 81863 | chlo |
| PpPPR53 | 6.27 | 117209 | cyto |  | PpPPR118 | 6.21 | 93468 | chlo |
| PpPPR54 | 6.06 | 100505 | chlo |  | PpPPR119 | 8.79 | 81176 | cyto |
| PpPPR55 | 6.76 | 51836 | cyto |  | PpPPR120 | 7.78 | 78668 | nucl |
| PpPPR56 | 8.62 | 90777 | chlo |  | PpPPR121 | 7.34 | 70384 | chlo |
| PpPPR57 | 7.97 | 69273 | chlo |  | PpPPR122 | 7.13 | 70544 | mito |
| PpPPR58 | 6.33 | 70310 | chlo |  | PpPPR123 | 7.52 | 76795 | chlo |
| PpPPR59 | 7.64 | 34493 | chlo |  | PpPPR124 | 6.32 | 77195 | chlo |
| PpPPR60 | 7.14 | 99789 | chlo |  | PpPPR125 | 6.67 | 85368 | chlo |
| PpPPR61 | 6.88 | 72891 | nucl |  | PpPPR126 | 6.83 | 100188 | mito |
| PpPPR62 | 6.45 | 106529 | chlo |  | PpPPR127 | 8.04 | 72664 | chlo |
| PpPPR63 | 7.09 | 64386 | chlo |  | PpPPR128 | 6.44 | 74128 | mito |
| PpPPR64 | 7.16 | 84602 | chlo |  | PpPPR129 | 6.24 | 85515 | chlo |
| PpPPR65 | 7.57 | 84724 | chlo |  |  |  |  |  |

pI: isoelectric point; mw: molecular weights; chlo: chloroplast, mito: mitochondria; cyto: cytoplasm; nucl: nucleus; vacu: vacuole; plas: plasma membrane.

Table S2 The Ka and Ks value of collinear genes

| Gene1 | Gene2 | Ka | Ks | Ka/Ks |
| --- | --- | --- | --- | --- |
| PpPPR2 | PpPPR119 | 0.02 | 0.05 | 0.39 |
| PpPPR48 | PpPPR24 | 0.03 | 0.20 | 0.17 |
| PpPPR51 | PpPPR19 | 0.10 | 0.23 | 0.45 |
| PpPPR57 | PpPPR16 | 0.07 | 0.11 | 0.64 |
| PpPPR59 | PpPPR10 | 0.09 | 0.16 | 0.56 |
| PpPPR64 | PpPPR65 | 0.01 | 0.02 | 0.53 |
| PpPPR67 | PpPPR12 | 0.06 | 0.30 | 0.18 |
| PpPPR70 | PpPPR87 | 0.07 | 0.21 | 0.34 |
| PpPPR74 | PpPPR116 | 0.10 | 0.29 | 0.33 |
| PpPPR77 | PpPPR114 | 0.10 | 0.27 | 0.37 |
| PpPPR80 | PpPPR30 | 0.58 | 2.09 | 0.28 |
| PpPPR94 | PpPPR5 | 0.04 | 0.18 | 0.25 |
| PpPPR99 | PpPPR6 | 0.05 | 0.26 | 0.19 |
| PpPPR100 | PpPPR7 | 0.06 | 0.14 | 0.46 |
| PpPPR102 | PpPPR8 | 0.11 | 0.30 | 0.38 |
| PpPPR102 | PpPPR103 | 0.43 | NaN | NaN |
| PpPPR104 | PpPPR9 | 0.02 | 0.18 | 0.14 |
| PpPPR105 | PpPPR38 | 0.22 | 0.65 | 0.33 |
| PpPPR106 | PpPPR38 | 0.04 | 0.33 | 0.12 |
| PpPPR110 | PpPPR39 | 0.04 | 0.18 | 0.21 |
| PpPPR111 | PpPPR39 | 0.04 | 0.18 | 0.21 |
| PpPPR120 | PpPPR40 | 0.04 | 0.24 | 0.16 |
| PpPPR121 | PpPPR122 | 0.01 | 0.02 | 0.44 |
| PpPPR123 | PpPPR42 | 0.06 | 0.18 | 0.34 |
| PpPPR124 | PpPPR45 | 0.03 | 0.16 | 0.19 |

Table S3 The structural variation of DYW-PPR genes

| Type | Gene | Ref | Alt | QUAL | CL | CM |
| --- | --- | --- | --- | --- | --- | --- |
| nonsyn-SNP | PpPPR46 | T | C | 196.34 | T/T:116,0:116 | T/C:75,14:89 |
| nonsyn-SNP | PpPPR49 | G | A | 238.91 | G/G:47,0:47 | G/A:72,14:86 |
| nonsyn-SNP | PpPPR54 | C | A | 1010.34 | C/C:132,0:132 | C/A:60,29:89 |
| nonsyn-SNP | PpPPR54 | C | T | 1010.34 | C/C:132,0:132 | C/T:60,29:89 |
| nonsyn-SNP | PpPPR54 | T | C | 931.34 | T/T:131,0:131 | T/C:65,27:92 |
| nonsyn-SNP | PpPPR61 | C | T | 288.91 | C/C:42,0:42 | C/T:77,14:91 |
| nonsyn-SNP | PpPPR61 | C | T | 805.91 | C/C:42,0:42 | C/T:58,24:82 |
| nonsyn-SNP | PpPPR61 | G | T | 734.91 | G/G:42,0:42 | G/T:60,22:82 |
| nonsyn-SNP | PpPPR61 | G | A | 608.91 | G/G:42,0:42 | G/A:60,19:79 |
| nonsyn-SNP | PpPPR61 | A | G | 623.91 | A/A:42,0:42 | A/G:55,19:74 |
| stopgain | PpPPR61 | G | A | 539.91 | G/G:42,0:42 | G/A:55,17:72 |
| nonsyn-SNP | PpPPR75 | A | C | 371.91 | A/A:44,0:44 | A/C:69,14:83 |
| nonsyn-SNP | PpPPR75 | A | C | 371.91 | A/A:44,0:44 | A/C:69,14:83 |
| nonsyn-SNP | PpPPR75 | C | A | 1959.92 | C/C:69,0:69 | C/A:52,52:104 |
| nonsyn-SNP | PpPPR75 | C | A | 1837.91 | C/C:60,0:60 | C/A:49,48:97 |
| nonsyn-SNP | PpPPR75 | A | C | 2197.91 | A/A:60,0:60 | A/C:53,58:111 |
| nonsyn-SNP | PpPPR75 | T | C | 2159.91 | T/T:60,0:60 | T/C:53,57:110 |
| nonsyn-SNP | PpPPR75 | T | C | 2140.91 | T/T:60,0:60 | T/C:51,55:106 |
| nonsyn-SNP | PpPPR75 | T | C | 2137.91 | T/T:60,0:60 | T/C:52,55:107 |
| stopgain | PpPPR75 | G | T | 1350.34 | G/T:64,39:103 | G/G:107,0:107 |
| nonsyn-SNP | PpPPR85 | T | G | 1046.91 | T/T:113,0:113 | T/G:132,38:170 |
| nonsyn-SNP | PpPPR85 | A | G | 487.91 | A/A:102,9:111 | A/G:84,20:104 |
| nonsyn-SNP | PpPPR85 | C | T | 336.91 | C/C:112,8:120 | C/T:72,19:91 |
| nonsyn-SNP | PpPPR91 | T | G | 72.91 | T/T:29,0:29 | T/G:29,4:33 |
| nonsyn-SNP | PpPPR92 | T | C | 2646.34 | T/T:146,0:146 | T/C:91,73:164 |
| nonsyn-SNP | PpPPR92 | C | T | 561.34 | C/C:114,0:114 | C/T:63,19:82 |
| nonsyn-SNP | PpPPR92 | A | C | 849.74 | A/A:94,0:94 | A/C:50,24:78 |
| nonsyn-SNP | PpPPR92 | A | T | 1027.34 | A/A:94,0:94 | A/T:47,28:75 |
| nonsyn-SNP | PpPPR92 | A | G | 1024.34 | A/A:94,0:94 | A/G:48,28:76 |
| nonsyn-SNP | PpPPR92 | T | A | 1028.91 | T/T:52,0:52 | T/A:46,28:74 |
| nonsyn-SNP | PpPPR92 | C | A | 172.34 | C/C:93,0:93 | C/A:42,9:51 |
| nonsyn-SNP | PpPPR22 | G | A | 375.91 | G/G:63,4:67 | G/A:76,15:91 |
| nonsyn-SNP | PpPPR22 | T | C | 351.91 | T/T:64,0:64 | T/C:71,14:85 |
| frameshift | PpPPR75 | CA | C | 2354.88 | CA/CA:60,0:60 | CA/C:52,60:112 |
| frameshift | PpPPR75 | GCCAT | G | 2309.88 | GCCAT/GCCAT | GCCAT/G |
|  |  |  |  |  | :60,0:60 | :53,59:112 |
| nonframeshift | PpPPR92 | T | TAGC | 302.88 | T/T:110,0:110 | T/TAGC:64,12:76 |
| nonframeshift | PpPPR92 | A | ACCG | 849.7 | A/A:94,0:94 | A/ACCG:50,24:78 |
